# Structural and Viscoelastic Properties of Bacterial Cellulose Composites: Implications for Prosthetics

**DOI:** 10.3390/polym16223200

**Published:** 2024-11-18

**Authors:** Natalia Pogorelova, Daniil Parshin, Anna Lipovka, Alexey Besov, Ilya Digel, Pyotr Larionov

**Affiliations:** 1 Department of Food and Food Biotechnology, Omsk State Agrarian University, Omsk 644008, Russia; na.pogorelova@omgau.org; 2 Lavrentyev Institute of Hydrodynamics, Novosibirsk 630090, Russia; lipovkaai@yandex.ru (A.L.); besov@catalysis.ru (A.B.); 3 Institute for Bioengineering, FH Aachen—University of Applied Sciences, 52066 Aachen, Germany; digel@fh-aachen.de; 4 Novosibirsk Research Institute of Traumatology and Orthopaedics n.a. Ya.L. Tsivyan, Novosibirsk 630091, Russia; ptrl@mail.ru

**Keywords:** bacterial cellulose, polysaccharide composite, composite material, viscoelastic characteristics of materials, fibrillar structure, strength properties

## Abstract

This study investigates the morphological, mechanical, and viscoelastic properties of bacterial cellulose (BC) hydrogels synthesized by the microbial consortium *Medusomyces gisevii*. BC gel films were produced under static (S) or bioreactor (BioR) conditions. Additionally, an anisotropic sandwich-like composite BC film was developed and tested, consisting of a rehydrated (S-RDH) BC film synthesized under static conditions, placed between two BioR-derived BC layers. Sample characterization was performed using scanning electron microscopy (SEM), atomic force microscopy (AFM), rheometry, and uniaxial stretching tests. To our knowledge, this is the first study to combine uniaxial and rheological tests for BC gels. AFM and SEM revealed that the organization of BC fibrils (80±20 nm in diameter) was similar to that of collagen fibers (96±31 nm) found in human dura mater, suggesting potential implications for neurosurgical practice. Stretching tests demonstrated that the drying and rehydration of BC films resulted in a 2- to 8-fold increase in rigidity compared to other samples. This trend was consistent across both small and large deformations, regardless of direction. Mechanically, the composite (BioR+S-RDH) outperformed BC hydrogels synthesized under static and bioreactor conditions by approx. 26%. The composite material (BioR+S-RDH) exhibited greater anisotropy in the stretching tests compared to S-RDH, but less than the BioR-derived hydrogels, which had anisotropy coefficients ranging from 1.29 to 2.03. BioR+S-RDH also demonstrated the most consistent viscoelastic behavior, indicating its suitability for withstanding shear stress and potential use in prosthetic applications. These findings should provide opportunities for further research and medical applications.

## 1. Introduction

Traditionally, biopolymers have attracted scientific interest due to their potential applications in biomedicine. Implantable devices made from biopolymers like collagen, cellulose, and chitosan are compatible with animal cells and tissues. The unique 3D structures of scaffolds made from these polymers support cell growth. The well-controlled porosity and size of fibers, especially in cellulose (fibrils), facilitates cell infiltration, promotes vascularization, and enhances nutrient and waste exchange. This in turn enhances the strength of the extracellular matrix, aiding tissue formation without damage [1,2].

Research into the use of bacterial cellulose (BC) as part of hybrid artificial fabrics has demonstrated its effectiveness in enhancing both mechanical properties and biocompatibility. Specifically, a developed hydrogel, PVA-BC(polyvinyl alcohol–bacterial cellulose), not only mimicked the nonlinear mechanical characteristics of heart valves but also displayed anisotropic properties when subjected to loads [3,4]. However, composite materials that combine a polysaccharide matrix with artificial polymers may provoke adverse biological reactions.

Therefore, questions regarding the control of structural versus mechanical characteristics during the self-assembly of a fibrillar 3D structure of bacterial cellulose remain relevant. Interestingly, during BC biosynthesis, both acetic acid bacteria and cell-free enzyme systems can influence the architecture of the cellulose matrix [5,6,7]. The presence of oligosaccharides in the nutrient medium during the self-assembly stage of a BC 3D structure leads to a denser arrangement of fibrils and reduces the size and pore volume of the cellulose material. These changes result in significant advantages for the BC as a biomedical dressing material, manifesting as a decreased water absorption capacity and enhanced water retention during evaporation [8,9].

Some important alterations in the properties of BC hydrogels due to underlying physicochemical processes have been identified. Our previous study on the strength properties of BC gel films revealed notable shifts in their Young’s modulus, relative elongation, and tensile strength during their dehydration [10]. The dehydration of BC gel films using different methods may reduce the material’s stretching capacity by 10–20 times. Substantial changes were observed in terms of theoir strength and Young’s modulus values [11,12].

Currently, the analysis of their mechanical properties of using rheometric measurements offers a convenient method for characterizing biphasic materials of biological significance [13]. The dependence of the rheological properties of various polymers on their concentration [14] and age [15] is well known. For biocompatible polymers, it is critical to consider factors such as temperature, pH, biosynthesis parameters, etc., which dramatically change the load-bearing abilities of such materials. The influence of the bioreactor type on the mechanical properties of synthesized biomaterials has been recently thoroughly reviewed by Regonesi [16]. Despite the large number of works in the field of the strength and viscoelastic properties of hydrogels [17,18,19], the approaches used for testing remain controversial [20]; some approaches require significant revision both in their execution and during the interpretation of their data [21]. In this study, we performed SEM and AFM examinations, a series of uniaxial tests, and a dynamical mechanical analysis (DMA) of BC films, including those obtained under bioreactor conditions, to assess the difference in their strength and viscoelastic characteristics. The joint analysis of their strength and viscoelastic properties, as well as possible interpretations of their frequency test, are provided for the first time for this type of material and should contribute to their possible application in biomedicine.

## 2. Materials and Methods

### 2.1. Composition of the Nutrient Medium and the Process of Biosynthesis, Under Static Conditions, of BC(S) and BC(S-RDH)

The synthesis of bacterial cellulose was carried out under static cultivation conditions using the microbial consortium *Medusomyces gisevii*, as described in previous studies [11,22]. *Medusomyces gisevii* is a stable microbial community including 15–30 genera, which are predominantly yeasts (especially *Zygosaccharomyces* sp.) and acetic acid bacteria (primarily *Gluconacetobacter* sp. and *Acetobacter* sp.). The exact species composition of this culture is described in detail elsewhere [10,23,24]. The nutrient medium consisted of glucose (Sigma-Aldrich Co.) at a mass concentration of 7.5% dissolved in 0.4% regular green tea extract (Woodbury Sugar Shed Co., USA). The tea solution was prepared by putting 4 g of dried green tea in 1 L of hot (80 °C) tap water for 15 min, resulting in approximately 1.21 g/L of green tea extractives in the medium.

BC biosynthesis of *Medusomyces* was carried out in cube-shaped polypropylene tanks (height 15 cm × width 17 cm × length 10 cm) at a temperature of 25±2 °C for 8 days. The medium was inoculated with a suspension of *Medusomyces* biomass, previously adapted (for seven days) to a carbon source—glucose. The initial volume of the culture medium in the reservoir was 1.5 L, and the initial ratio of inoculum to culture medium was adjusted to 1:3 (*v*/*v*).

Active (titratable) acidity was not adjusted. The synthesized cellulose formed as a swollen gel film at the air/water interface, the thickness of which increased with increasing cultivation time. BC films at the air/liquid interface were separated from the culture liquid, gently squeezed, and washed with deionized water until neutral pH values (close to 7.0) were achieved. The washed material was incubated in the same volume of 0.1 M NaOH at 80 °C for 90 min to remove remaining microbial cells.

This step was repeated four times until a colorless transparent BC film was obtained, and then it was washed again with water to neutral pH values and pressed to remove water. The dry matter content of the samples was 1.4% (BC(S)). BC samples were dried under room conditions (temperature 25±2 °C, humidity 65 ± 1%) until a constant weight was obtained. Before testing for strength characteristics, the BC was rehydrated for 12 h in an isotonic sodium chloride solution. This material is hereinafter referred to as BC(S-RDH).

### 2.2. Preparation of Bacterial Cellulose Composite BC(BioR+S-RDH)

A laboratory drum-type bioreactor with a rotation speed of 8 rpm has been developed at the Omsk State Agrarian University department. In this reactor, a gel film of bacterial cellulose is formed on the surface of a rotating drum, which comes into contact with the culture medium, which has a volume *V* = 7.5 L at T=28 °C. After the biosynthesis process, the BC was removed from the drum in the form of a “sleeve” (BioR), cut, and examined in the same way as the samples of cellulose gel films obtained under static cultivation conditions (Table 1). BioR-H2O samples were partially dehydrated in the air to a cellulose content of 1.35%. The technical characteristics of the bioreactor are being patented (application filing date 1 August 2024).

Composite films were prepared by sandwiching a rehydrated cellulose sample BC(S-RDH) between two layers of the BC gel matrix synthesized under the reactor conditions. The preparation of the nutrient medium and seed material is the same as at the earlier stages. The resulting sample consisted of three layers of BC(BioR+S-RDH) of 3.2 mm thickness; the outer two layers were gel films synthesized under BC reactor conditions (BioR) and the middle layer was a rehydrated BC film synthesized under static conditions BC(S-RDH).

### 2.3. Scanning Electron Microscopy (SEM)

The primary study of the spatial orientation of BC microfibrils was carried out using the SEM method. For each experimental treatment, at least three samples, previously cut with a sharp blade into 2 × 2 mm pieces, were examined using a JCM 5700 scanning electron microscope (JEOL Ltd., Japan). Since the studied BC samples are dielectric, low accelerating voltages were used that did not exceed 5 kV. SEM images were acquired in a plane perpendicular to the surface of the BC film from at least three different randomly selected positions and using three different BC samples and magnifications ranging from ×500 to ×10,000. Quantitative data related to inclusion size, average pore size, and fiber arrangement were obtained using JCM 5700 image analysis software by determining the average of 20 individual measurements and the standard deviation for each measured parameter.

### 2.4. Atomic Force Microscopy (AFM)

The test using atomic force microscopy (AFM) was carried out on a scanning probe microscope NTEGRA Prima (NT-MDT, Russia) in semi-contact mode using HA_NC Etalon Series probe sensors with a characteristic stiffness of 3.5 N/m. During the scanning process, a mismatch signal was also recorded in the feedback circuit. It indicates the deviation of the interaction parameter between the probe and the sample when it moves to the nearest test point. The interaction parameter refers to the amplitude of the probe’s oscillation, recorded by measuring the difference in the photocurrent between the halves of the photodetector, which occurs due to the impact of laser radiation reflected from the probe sensor. As a result, the recorded parameter was measured in nA. Image processing was carried out using Gwyddion software, distributed under the GNU GPL license.

### 2.5. Dynamic Mechanical Analysis (DMA)

**Figure 1:**
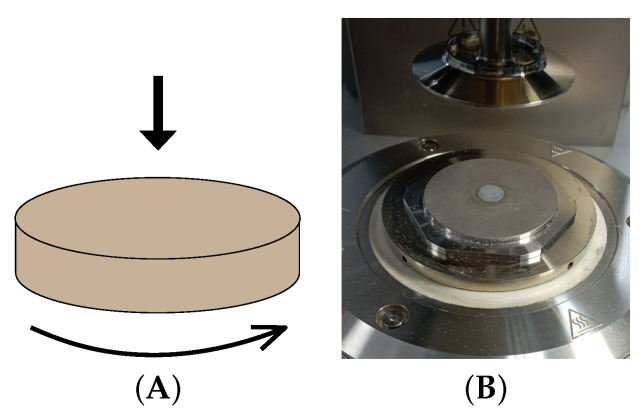
Schematic set-up used for the measurement of the viscoelastic characteristics of the samples (**A**); installation of a BC hydrogel sample prior to testing on rheometer (**B**).

For performing viscoelastic DMA tests with BC films, a circle with a diameter of 10 mm was cut out using a stainless steel matrix. Samples were taken from the same part of the gel film as where samples were taken to perform strength tests. The circle was placed in the center of the lower substrate of the measuring system of an Anton Paar MCR302 rheometer (Figure 1). It is preferable to carry out the testing of samples at the target temperature of their use [25]. In our case, the intended application of these hydrogels was as implants in biomedicine, so the chosen test temperature was (37 °C ± 0.02).

A plane-to-plane measuring system (PP50/S) with a given roughness was used (Anton Paar GmbH, Germany). Then, the sample was loaded with a vertical force of 1 N, which ensured reliable contact of the sample with the measuring system. After clamping the sample, th rheometer system automatically determined its thickness as the gap between the upper and lower plates of the measuring system. We performed two types of rheometric tests: amplitude sweep and frequency tests. During the amplitude sweep test, the magnitude of the shear deformations varied and values of the loss and storage moduli were calculated. The frequency test determined the loss modulus and storage modulus at a given strain rate (10%) with respect to a frequency of deformation, which varied in the range of 0.01 to 1000 Rad$\cdot s^{-1}$. The aim, while performing amplitude sweep and frequency tests, was to achieve an accuracy of a 0.2% (default value) deviation from the mean value of the measurements performed. The rheometer automatically takes the required number of measurements and proceeds to the next test when achieving the above accuracy. The duration of one measurement series for each shear rate value was 20–30 s. Measurements were also made in the range of even smaller deformations (0.01–0.1%), but the accuracy of the measurements was not higher than 1–2%, and these data were not used in the analysis of the results. Further, the same thickness data were used in calculating the ultimate values of stress and Young’s moduli of the samples (see Section 2.7). Throughout all tests, the shear rate of the rheometer was controlled, which ensured the stable and constant contact of the sample with the measuring system. Amplitude and frequency tests were performed alternatingly. On pre-selected samples, the independence of the sequence of the tests was shown (first amplitude and then frequency, and vice versa). Each measurement is the average of 20 consecutive tests. The range of the deformation amplitude was from 0.1% to 20%, and the range of the deformation frequencies was from 0.1 to 100 Hz at a fixed deformation amplitude of 10%. The sequence of tests was established in this order because in order to conduct a frequency test it is necessary to know the range of the material’s linear response to shear deformation.

### 2.6. Uniaxial Stretching

**Figure 2:**
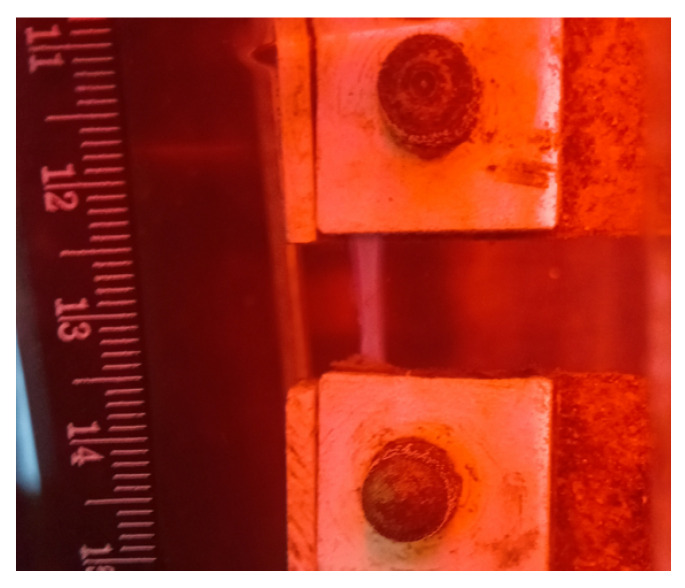
Sample during uniaxial stretching.

To carry out mechanical testing, rectangular samples were cut. Their exact geometry was then measured using graph paper, calipers, and a digital microscope. The samples were secured in clamps specially made for the tensile testing of biological tissues (Figure 2).

The clamps are made of plastic and wrapped with waterproof sandpaper to prevent slipping. The removable part of the clamps was secured with screws, firmly fixing the sample, and the fixator kept both parts stationary relative to each other, preventing damage to the sample during fastening. The clamps holding the fixed sample were placed in the jaws of the tensile testing machine, the fixator was removed and then, before the start of the test, the “Sample Protection” mode was turned on, which made it possible to avoid induced or accidental impacts on the sample.

During the entire test period, the cellulose sample was immersed in a physiological solution heated to a temperature of 37 °C.

Cyclic loading was performed with displacement control during loading and force control during unloading. The loading speed was 2 mm/min, and the step between cycles was 0.25 mm.

All mechanical tests were performed on a universal testing machine, Instron 5944 (Instrin, USA). In contrast to [26], a 250 N clamping system with a 10 N force sensor (measurement accuracy 0.5%) was used, along with a thermostatic bio-bath, at the Lavrentyev Institute of Hydrodynamics SB RAS. During the test, the samples were subjected to step-by-step stretching, following the concept of the preconditioning technique [27], with the absolute strain increased in steps of 0.25 mm, as well as with a first step of 0.25 mm and a strain rate of 2 mm/min. The thickness of the samples was assumed to be the same along their entire length and was measured for each of the samples during the rheometric tests (see Section 2.5). To perform strength tests, the samples were secured in jaws as shown in Figure 2 [28,29]. Sample rupture occurred in the middle zone between the clamps. For all types of BC hydrogel films—S, S-RDH, BioR, BioR+S-RDH, and BioR-H2O (Table 1)—rectangular samples were cut in two directions that were orthogonal to each other.

### 2.7. Calculation of Anisotropy Coefficient

There are various approaches to the synthesis of non-isotropic materials based on hydrogels [30]. In this case, the isotropy characteristic can be significant for the purposes of using hydrogels in medical applications [31]. Due to the fact that BC hydrogels are quite thin, and the redistribution of their reinforcing fibrils occurs in the material layer, the anisotropy coefficient *R* can be calculated using the formula for orthotropic materials [32]:

(1) $$R=\varepsilon_{dir1}/\varepsilon_{dir2},$$

where

(2) $$\varepsilon_{dir_{1,2}}=\ln\sigma_{dir_{1,2}}^{ultimate}.$$

$\sigma_{dir_{1,2}}^{ultimate}$ is ultimate strength value for each of the directions tested.

## 3. Results

### 3.1. SEM Data

The SEM analysis of biomedical materials’ ultrastructure enables a better prediction of their clinical effectiveness

**Figure 3:**
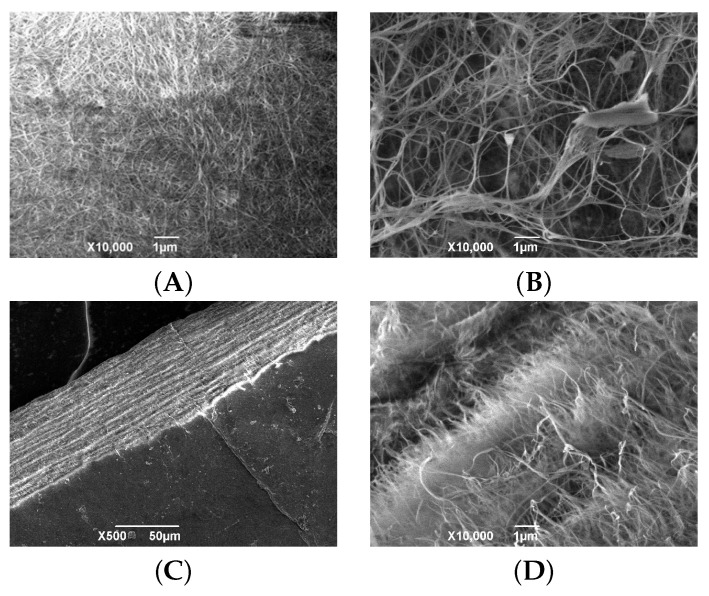
Visualization of the differences between the sides of the BC(S) film and its cross section: SEM images of the BC film synthesized under static conditions (**A**); the denser side of the surface is that formed at the air–nutrient interface. (**B**) The porous opposite side. (**C**,**D**) Layered structure seen in the cross section of the sample.

In the SEM image of BC(S) (Figure 3B), the fibrils are arranged chaotically, with a large number of spaces between them. This arrangement of fibrils leads to the formation of pores of different diameters on the surface and throughout the entire matrix of the BC samples.

In comparison with stationary cultivation, BC gel films from a bioreactor are characterized by greater transparency and a −0.5% lower amount of dry substances, which is confirmed by the greater sparseness of the fibrillar network in the AFM images of the BC(BioR) samples in comparison with the BC(S) ones (Figure 3B). The BC(BioR) structure is characterized by spatially oriented, parallel cellulose fibers, which may determine the anisotropy of this material.

**Figure 4:**
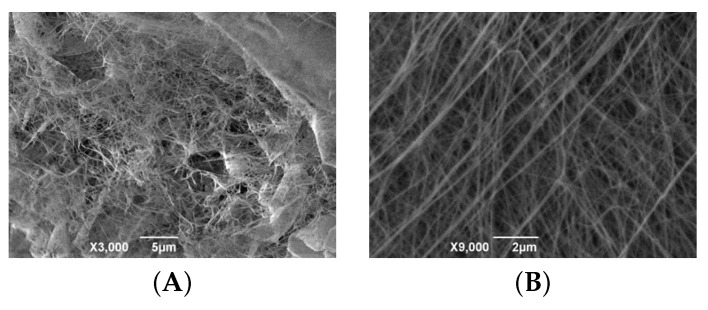
SEM images of lyophilized BC(BioR) film: (**A**,**B**) porous structure of a section of some samples.

The SEM images of a cross section of BC synthesized under reactor conditions (Figure 4) also revealed loosely spaced fibrils with abundant empty spaces throughout the BC(BioR) polymer matrix. It is interesting to note the absence of a layered structure in BC synthesized in a drum-type bioreactor in comparison with BC(S) samples synthesized under static conditions.

### 3.2. AFM Data

**Figure 5:**
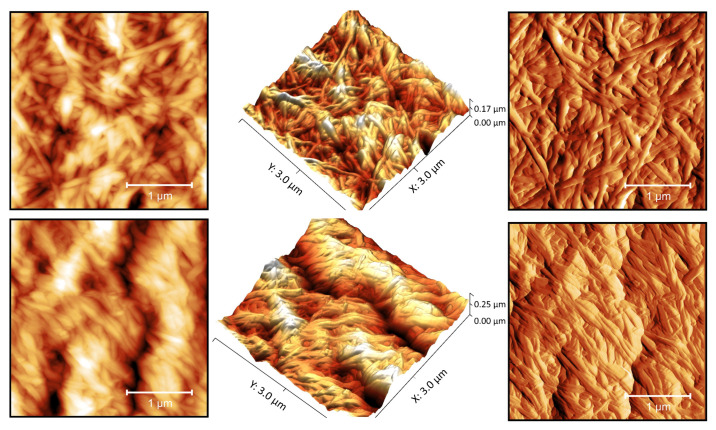
AFM photographs of the surface of BC samples synthesized under static (**top row**) and reactor (**bottom row**) conditions: 2D image (**top left**, **bottom left**); 3D image (**top center**, **bottom center**); and differential signal (**top right**, **bottom right**), respectively.

BC, synthesized both under static and reactor conditions, had a clearly defined fibrillar structure (Figure 5); the rectilinear nature of its cellulose fibers, the length of which is greater than the visible field (>5 μm), is to be noted. BC(BioR) cellulose samples are characterized by a directional orientation to their parallel fibers, in comparison with BC(S) samples.

The average diameter of BC fibrils is 80±20 nm and their dispersion is comparable to those of collagen fibers of the human dura mater (DRM) −96±31 nm, which makes BC film gel a promising material in neurosurgical practice. Significantly larger fiber sizes come from artificial materials with a fibrous structure, and these are currently offered for the plastic surgery of dura mater defects, such as Duraform (Codman, USA), Belkozin (JSC Belkozin, RF), Lyostypt (BBraun, Germany), Durepair (Medtronic, USA), Lyoplant (BBraun, Germany), Neuropatch (BBraun, Germany), Gore Preclude dura substitute (USA), and ReDura (Medprin, China) [33,34].

**Figure 6:**
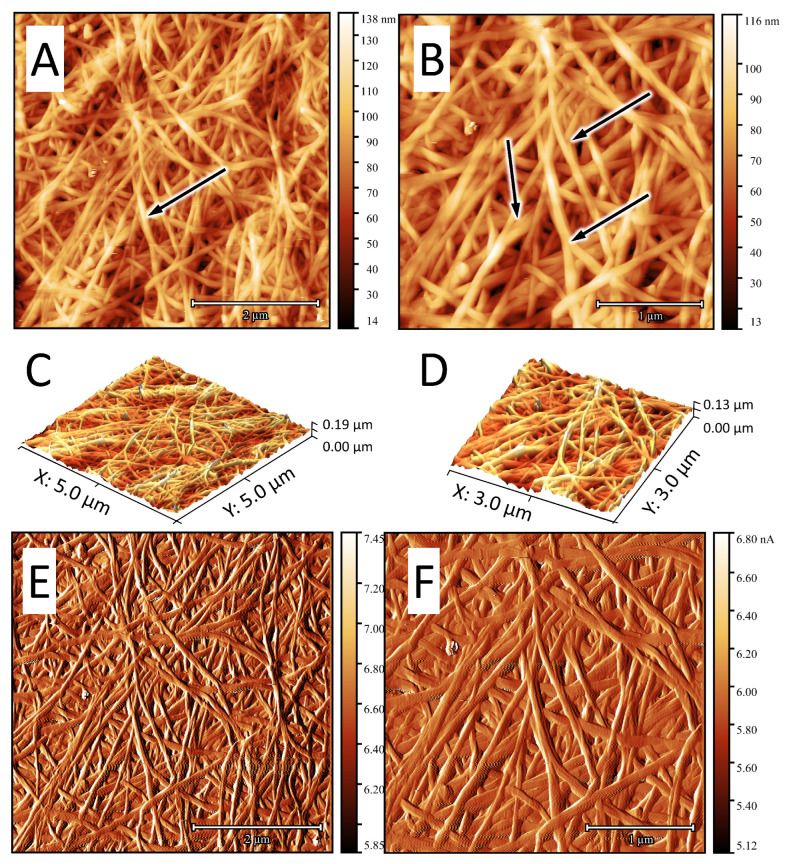
AFM photographs of the surface of a lyophilized BC gel film synthesized under reactor conditions: 2D image (**A**,**B**); 3D image (**C**,**D**); and differential signal (**E**,**F**), respectively. Arrows indicate branching points of BC fibrils. Scan size: 5 × 5 μm (**left**); 3 × 3 μm (**right**).

A study of lyophilized samples—BC gels, which to a greater extent retain the native state of the cellulose fibers of hydrogels—showed a sparse fibrillar structure in comparison, which confirms the data of our SEM studies. Cellulose fibers of lyophilized samples are characterized by a greater crimp. The size of the free spaces between fibrils is highly variable and reaches 1.5 microns. The strength characteristics of BC are largely determined by the superstructural organization of its fibrillar cellulose matrix. Two characteristic types of interaction between fibrils have been identified: branching points, where one fiber is divided into two (Figure 6, indicated by arrows) and contact points, determined by the spatial interaction of fibrils and the occurrence of hydrogen bonds and/or van der Waals forces.

Presumably, the branch points determine the elasticity of the BC films and the contact points determine the solidly similar behavior of the cellulose matrix [35]. Since anisotropic hydrogel membranes have great potential in tissue engineering and bioseparation, one of the objectives of this study was to create an anisotropic BC hydrogel film with increased strength characteristics, synthesized under the conditions of our developed drum-type bioreactor. To do this, during the biosynthesis of BC under reactor conditions, BC(S-RDH) was introduced into a hydrogel synthesized over 4 days and then the biosynthesis was continued; within 8 days the three-layer structure of a composite material BC(BioR+S-RDH) was generated.

### 3.3. Strength Test Results

**Figure 7:**
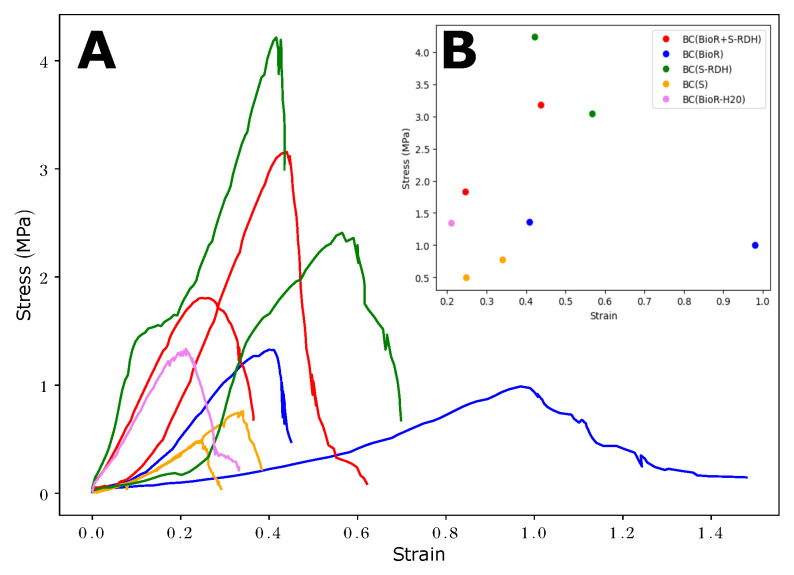
Overlapping strain–stress diagrams of uniaxial strength tests (**A**) and samples’ corresponding ultimate stress and ultimate strain values (**B**).

Native BC(S) and BC(BioR) BC samples have comparable stress–strain curve shapes during testing. The curve showed an increased slope depending on the deformation direction after 7% and 22% for BC(BioR) samples synthesized in the bioreactor and about 8% for BC(S) obtained under static conditions, which corresponds to the region of the more elastic deformation of cellulose fibrils, as seen in Figure 7. The low-deformation region corresponds to the rearrangement, sliding, straightening, and orientation of BC fibrils in the direction of extension. The results of strength tests show that the dehydration of BC hydrogels significantly increases the strength characteristics of these samples BC(S-RDH) in comparison with never-dried BC samples (hydrogels). This tendency is observed at small and ultimate deformations regardless of the deformation direction. Interestingly, the isotropy of cellulose material and the resulting composite is significantly affected by the BC biosynthesis method and, to a lesser extent, thermal dehydration. Thus, from Table 2, it can be seen that the samples synthesized under static conditions, rehydrated BC(S-RDH) and BC(S) hydrogel, have the lowest anisotropy with respect to deformations (Table 2).

The composite material BC(BioR+S-RDH), exhibits greater anisotropy in the tensile test in comparison with the samples of cellulose hydrogels synthesized under static conditions, but less with BC(BioR) hydrogels produced in a drum-type bioreactor. This is most likely due to the method of the placement of the rehydrated BC(S-RDH) sample composite matrix inro the BC(BioR) structure. A significant difference in elasticity during tests in different directions is characteristic of BC(BioR) gel films synthesized in a bioreactor, whereas for rehydrated BC(S) and composite BC(BioR+S-RDH) gel films, anisotropy is determined for both their stress and strain. During the tensile testing of BC gel films, the configuration of their polymer matrix and the interaction of the polymer with water change is the initial stage. The ordered alignment of BC macrofibrils should reduce their resistance to deformation, but the isotropic fibrillar network effectively increases the rigidity of BC gel films synthesized under static BC(S) conditions. In addition, tensile stress apparently leads to fiber aggregation, which leads to a denser BC hydrogel structure. It is expected that the orientation of BC fibrils in space and their number in a volume will determine the properties of gel films during their deformation stretching. The rigidity of rehydrated BC(S-RDH) samples is 2–8 times higher compared to both BC(S) and BC(BioR) samples, which is probably largely determined by the aggregation of fibrils during BC(S-RDH)’s dehydration and the smaller amount of native BC gel films synthesized under static and reactor conditions [13]. In [36], a difference in the apparent value of the Young’s modulus for BC hydrogels produced from bacterial strains was established.

### 3.4. DMA Test Results

**Figure 8:**
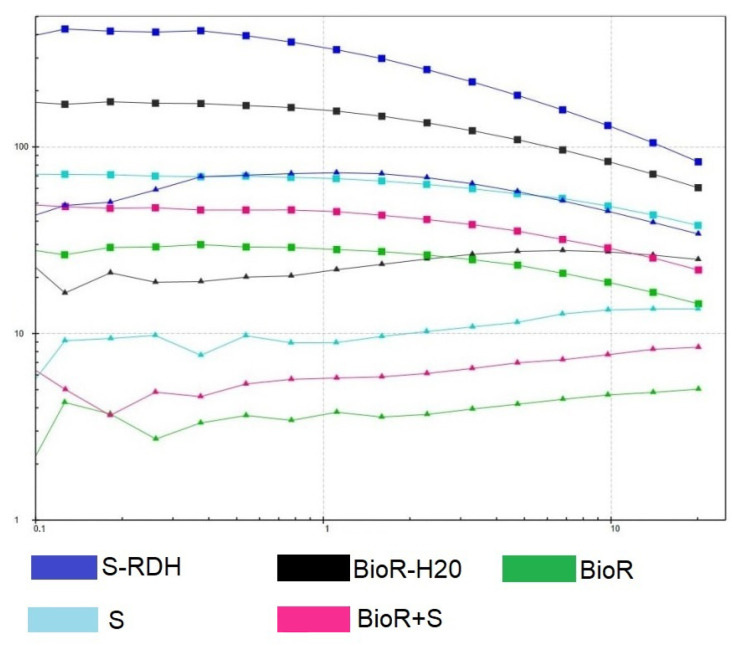
Amplitude sweep data of tested BC hydrogel samples. Squared line—$G^{\prime }$. Triangled line—$G^{\prime \prime }$ data (Pa). Horizontal axis—shear strain (%).

The results of the amplitude sweep test (Figure 8) showed that at low shear stresses, the strongest samples were BC(S-RDH) and BioR-H2O. Moreover, BC(S-RDH) had an elastic modulus that is an order of magnitude greater than BioR-H2O, the elastic modulus of which is, in turn, an order of magnitude higher than that of BC, e.g., the S, BioR+S-RDH, and BioR types (Table 3).

**Figure 9:**
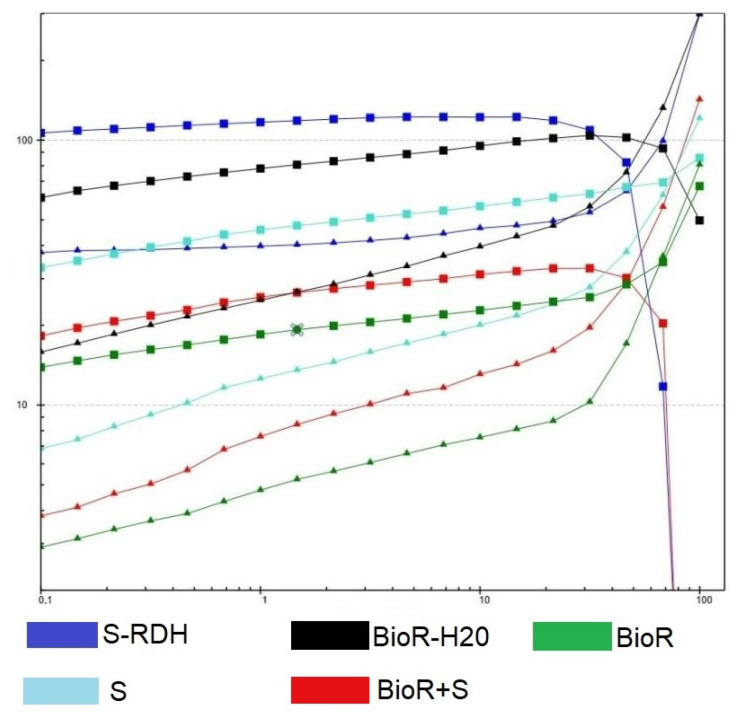
Frequency sweep data of tested hydrogel samples. Squared line—$G^{\prime }$ (storage modulus). triangled line—$G^{\prime \prime }$ (loss modulus) data (Pa). Horizontal axis—angular frequency (1/s).

As expected, the introduction of rehydrated BC(BC(S-RDH)) into the BC(Bioreact) hydrogel matrix increased the elastic properties of the material as a whole: throughout the entire studied section the deformation value changed from 0.1% to 20%; the superiority of the BioR + S curve over BioR is visible. However, with an increase in the deformation amplitude and upon reaching approximately 1%, the most elastic samples (S-RDH and BioR-H2O) begin to exhibit nonlinearity and degrade significantly up to the limiting deformation mode. The remaining samples show significant stability in the range of up to 3% deformation, after which nonlinear effects begin to appear, but the nature of the degradation of their strength characteristics is significantly more stable compared to the most elastic samples. The composite sample BC(BioR + S-RDH) demonstrates the most monotonous change in viscoelastic characteristics, most likely as a result of morphological features, i.e., its layer-by-layer composition, where each layer to a certain extent reacts differently to shear stresses, while its shear deformations themselves are different layer by layer. This morphology of the BC composites significantly affects the results of their strength and shear tests. The results of the frequency test (Figure 9) show that the most stable samples are BC(S), BioR, and BC(BioR-H2O), which have the highest frequency “flow points”—intersections of two curves corresponding to the values of their elasticity and loss moduli.

At present, there is no unambiguous interpretation of such an intersection. However, the change in the position of the curves relative to each other when passing this point indicates that if the elastic component prevailed at a lower deformation frequency, then with an excess of a certain deformation frequency, the viscous component begins to prevail. High frequencies are not typical physiological conditions for a living organism. It can be stated that the intersection point of the curves of these materials at high frequencies corresponds to their stability in the sense of their use in biomedical applications. On the contrary, the occurrence of an intersection of the curves at low frequencies (up to 16 Hz) corresponds to a high probability of achieving stability under physiological conditions, which corresponds to the unstable behavior of the material. In addition, a “strengthening” behavior of the BC(S) and BioR materials is visible with an increase in the deformation frequency, while the other samples began to significantly lose their elastic properties with an increase in the deformation frequency in the range of 20–160 Hz.

## 4. Discussion

Bacterial cellulose hydrogels are a popular biocompatible material for the manufacture of implants. They can be used independently or filled with patient stem cells when installing dura mater implants [37], menisci [38], and other cartilage [39] and vascular grafts [40,41]. However, for the appropriate use of certain types of hydrogels, it is necessary that at least their strength and viscoelastic characteristics correspond to the conditions of the organ tissues they replace. The mismatch of uniaxial and shear stresses in vivo for such implants can lead to their abnormal endothelialization [42,43]. This justifies, in particular, the interest of this study in the structural and strength properties of synthesized BC hydrogels, as well as in the various approaches to testing their strength and viscoelastic characteristics.

The discharged fibrillar structure of the BC gel film samples synthesized in the reactor should be noticed in comparison to those formed under static biosynthesis conditions. It is interesting that BC(S) is characterized by a layered structure, seen in its cross section, while this is absent for the BC(BioR) samples. The spatial orientation of BC hydrogel fibrils is determined by both SEM and AFM methods for lyophilized BC(BioR) samples, which makes this biosynthesis method promising in the production and design of anisotropic materials. The application of the assessment of mechanical properties and anisotropy of the materials for BC in particular has already taken place in the literature [44]. However, in that work, the authors did not test the samples in different directions and, in this case, it was permissible to talk only about morphological anisotropy. In our work, on the contrary, a similar analysis is carried out. In the work [45], the authors analyzed the change in the microstructure of the fiber orientation during the stretching of the samples. However, this analysis was performed at laboratory temperature (25 °C), which makes it impossible to apply the results of such an analysis to biomedical applications. In the work [46], the deformations of BC samples were assessed; however, the tests were performed in air, which makes the use of such results unsuitable for biomedical applications. In general, all authors note to one degree or another the anisotropy of the structure of BC films and the question remains as to the degree of anisotropy. Taking into account the anisotropy of vascular wall structures [47,48,49] and, conversely, the isotropy of the DM (dura mater)’s structure [50], it follows that the samples we studied are suitable to varying degrees for application either as vascular implants (for BC films with a high anisotropy coefficient) or as DM implants (for BC films with a low anisotropy coefficient). It is noteworthy that these works did not derive a specific anisotropy coefficient for types of artery or the dura mater. In addition to purely morphological similarities, this approach to determining the industrial purpose of using a BC film is determined by the loads (deformation characteristics) that are inherent in the replaced material. Thus, if the characteristic deformations for the dura mater material are within 1%, then the deformations of the vascular wall can normally reach 10–20%. It follows that when choosing a material as an implant, one should take into account not only the data on its ultimate loads and Young’s modulus, but also the nature of its curves in Figure 8. The steeper the graph, the earlier the material undergoes structural restructuring (the curve differs significantly from a linear one). A similar restructuring is described in detail for arterial tissue in [51] and this was confirmed experimentally [29]. The results obtained indicate that not all materials retain the linearity of their behavior even under deformations at the level of 1%.

**Figure A1:**
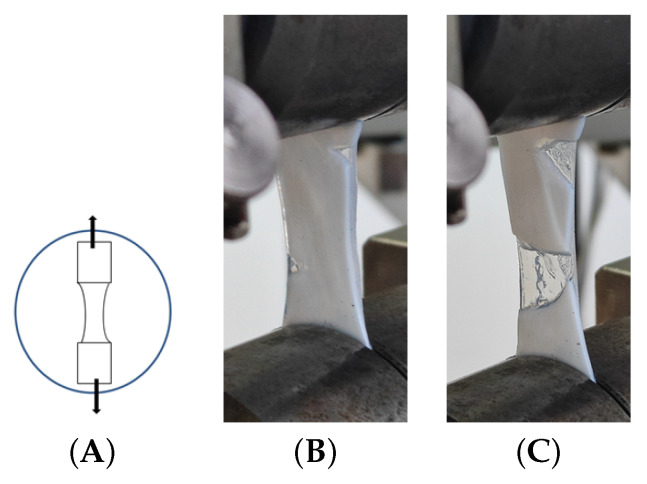
Visualization of BC composite rupture (BioR+S-RDH) in air: (**A**) test plan, (**B**) onset of rupture, and (**C**) the rupture of the middle layer occurs in the central part of the sample.

Despite the fact that tensile strength testing is the gold standard in the field of the strength of biomaterials [52,53], in our opinion, such a trend is impossible obtain without a detailed study of the area of interest in terms of the magnitude of the deformations of specific hydrogels. In addition, most of these tests are performed in air [20] (see Figure A1) rather than under physiological conditions. This approach significantly changes the strength characteristics of biomaterials and can lead to the destruction of an implant in vivo [54].

However, there are various approaches to strengthening BC hydrogels [55,56] using composites. In addition to their strength properties, such composites can also improve the biocompatibility properties of BC hydrogels [25,37]. The literature does not provide an accurate interpretation of the intersection points of the curves describing the dependence of the elastic modulus and losses as a function of the deformation frequency at a given amplitude, since the nature of such a point will significantly depend on the material. However, for materials for which the elastic modulus exceeds the loss modulus in a wide range of deformation amplitudes, understanding the fact that viscous losses begin to exceed the elastic modulus may indicate a violation of the structure of the studied BC samples at a given frequency. This effect is observed at a fairly high frequency (more than 30 Hz), which exceeds the physiologically relevant frequencies observed in the body and rather relates to deformations resulting from exposure to high-speed (and usually high-energy) impacts: road traffic accidents, exposure to damaging elements, etc. Similar rheological tests occur in the literature [57], but the authors do not pay attention to frequency tests. Meanwhile, due to the viscous structure of BC hydrogels, it is obvious that it is necessary to test the samples at different speeds at a given deformation amplitude (10%), which is achieved by varying the frequency. This result is new and has not previously been reported in the literature; however, the limiting factor is the lack of control over the structure of BC hydrogels during such a test. The development of such methods, in our opinion, would allow for significant progress in understanding the mechanics of cellulose hydrogels.

By jointly analyzing the data from strength tests on a universal tensile testing machine in a physiological solution at a temperature of 37 °C and on a rheometer (at the same temperature), we can note a direct correlation of the ultimate strength values of the samples with the curves in Figure A1. In addition, the amplitude test data set allows us to talk about the applicability of the studied samples to specific physiological processes for which this or that biomaterial is synthesized.

## 5. Conclusions

During mechanical tests, anisotropy was revealed to varying degrees for all the studied samples. The dependence of their mechanical properties on their biosynthesis conditions is shown via a static method and production in a drum-type reactor. When testing for uniaxial tension in different directions, a significant difference in elasticity is characteristic for hydrogels synthesized in a bioreactor compared to rehydrated and composite gel films. Cellulosic materials were obtained after rehydration and the introduction of rehydrated samples: the rehydration of BC(S-RDH) and BC(BioR+S-RDH) into BC hydrogels is marked by their greater strength and elasticity compared to the other samples, as well as the greater stability of their structure under large deformations compared to the strongest (rehydrated) gel film samples. Composite-material BC has a more stable structure in the face of shear and tangential stresses. Together with its more anisotropic properties relative to its ultimate deformation, this makes it more suitable than other studied samples for the manufacture of prostheses such as vascular tissues. In this work, a physical interpretation of the intersection point of the curves of the dependence of elastic moduli on the deformation frequency measured in a frequency test was given for the first time, and the data from an amplitude rheometric test were compared for the first time with the data from a strength uniaxial test performed under conditions close to physiological ones.

## Figures and Tables

**Table 1:** Different types of BC films used in this study.

Material	Synthesis Method	Notes
S	Hydrogel BC synthesized under static conditions	Dry matter content 1.40
S-RDH	Rehydrated sample S	
BioR	BC hydrogel obtained under reactor conditions	Dry matter content 0.85
BioR-H2O	Partially dehydrated hydrogel BioR	Dry matter content 1.35
BioR+S-RDH	Composite three-layer material BC, obtained under reactor conditions; the middle layer is rehydrated hydrogel BC S-RDH	

**Table 2:** The ultimate strength characteristics of BC hydrogel film samples, as well as their characteristics after small deformations during uniaxial testing in orthogonal directions (standard deviation is given in the brackets for 3 specimens of each material).

Value/ Material	S	S-RDH	BioR	BioR+S-RDH	BioR-H2O
Young modulus direction 1	1.28	5.14	9.51	2.74	5.67
Young modulus direction 2	1.23 (±0.14)	14.3 (±1.4)	4.33 (±0.67)	9.51 (±1.30)	
Ultimate stress direction 1 (MPa)	0.77 (±0.07)	2.43 (±0.34)	1.36 (±0.28)	3.17 (±0.78)	1.34 (±0.29)
Ultimate strain direction 1	0.34 (±0.04)	0.57 (±0.06)	0.4 (±0.05)	0.44 (±0.07)	0.21 (±0.03)
Ultimate stress direction 2 (MPa)	0.49 (±0.06)	4.23 (±0.82)	0.997 (±0.09)	1.83 (±0.26)	
Ultimate strain direction 2	0.25 (±0.03)	0.42 (±0.06)	0.98 (±0.08)	0.25 (±0.03)	
R-anisotropy	1.309 (±0.124)	1.288 (±0.1661)	2.03 (±0.218)	1.632 (±0.324)	

**Table 3:** Results of the DMA tests of different types of BC films. Each value in lines 3–8 has a 0.2% standard deviation value, as mentioned in Section 2.5. The data in 1–2 lines were obtained at the intersection of the interpolated curves of frequency–stress dependence.

Value/ Material	S	S-RDH	BioR	BioR+S-RDH	BioR-H2O
“Flow point”-Hz 1	69	46	53	46	53
“Flow point”-modulus (Pa) 2	70	64	97	30	97
El. mod. 0.1%	0.09	0.5	0.03	0.06	0.2
El. mod. 1%	0.75	34	0.3	0.5	17.4
El. mod. 3%	6	71	0.8	12.5	4.1
El. mod. 5%	30.3	90	9.5	17.5	57
El. mod. 10%	49	13.5	19	29.1	86
El. mod. 20%	80	18	30.5	4.7	13

**Figure A2:**
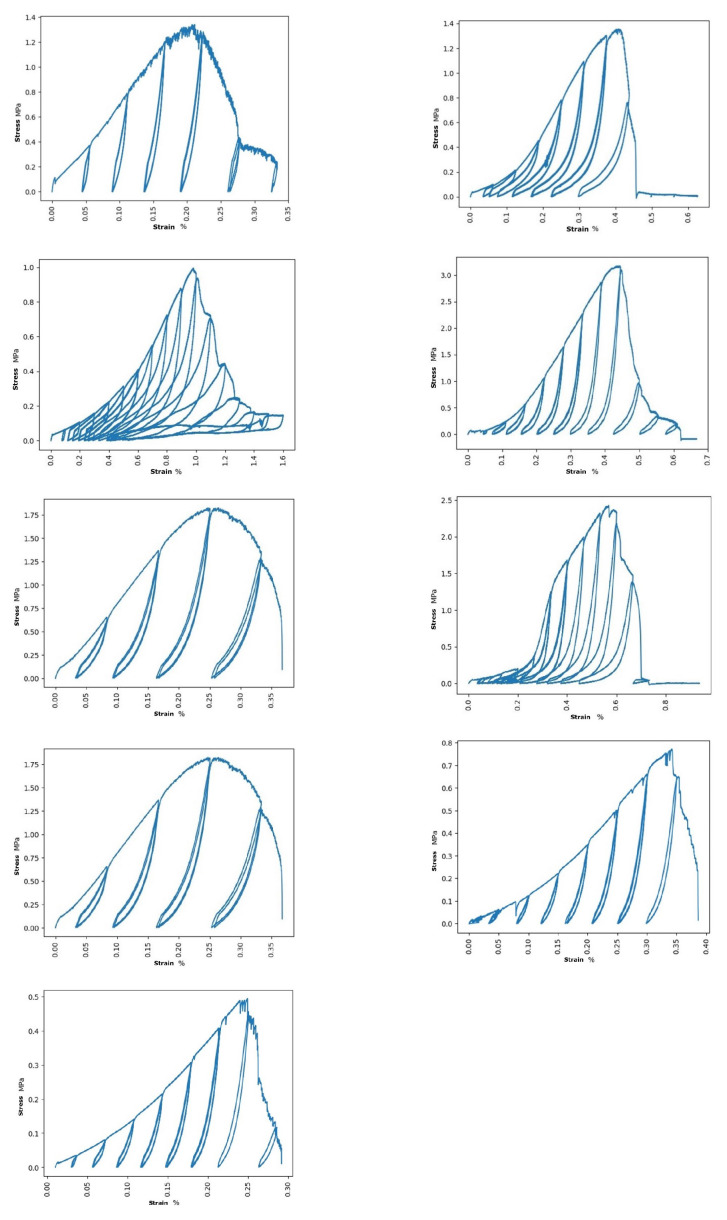
Full stress–strain diagrams of tensile tests.

## Data Availability

The research data from the uniaxial test are fully presented in the Appendixes Appendix A and Appendix B for one specimen of each of the BC materials. The full DMA protocol is attached as Supplementary Material to the Article.

## Supplementary Materials

The following supporting information can be downloaded at https://www.mdpi.com/article/10.3390/polym16223200/s1.

## Appendix B. Strain–Stress Diagrams of Tests of Various Types of Films in Orthogonal Directions

For sample BC(BioR-H2O), the test failed in one direction. The delamination of the material occurred during the process of securing the sample in the clamps.
