# Supplementary material for: Structural and Viscoelastic Properties of Bacterial Cellulose Composites: Implications for Prosthetics
Source: Polymers (Basel). 2024 Nov 18;16(22):3200. doi: 10.3390/polym16223200 (PMC11597974; doi:10.3390/polym16223200)
Supplement: Supplementary file 1 [file polymers-16-03200-s001.zip › Cell_bioreact_o┤_37_o│_PP50_S_oΘo╤oπ_oΣo╓_0,1_100_oñoΦ_o╘o╤o▐_10%_F_0_25N_22_09_23__12_11_13.pdf]

Company:
Street:
City:

Report

Test | Info Cell_bioreact_T_37_C_PP50_S_час_те_0,1_100_Гц_гам_10%_F_0_25N_22_09_23_

Test created by operator: temp
Test creation date: 22.09.2023 11:39:42

Origin of project:

Rheometer: MCR 302 SN82961886
Measuring System: PP50/S SN79497

-

Sample | Info

Sample name:
Batch No.:

Description:

-

Result Data

Viscosity | 1st point:
Viscosity | last point:

Regression:
Interpolation:

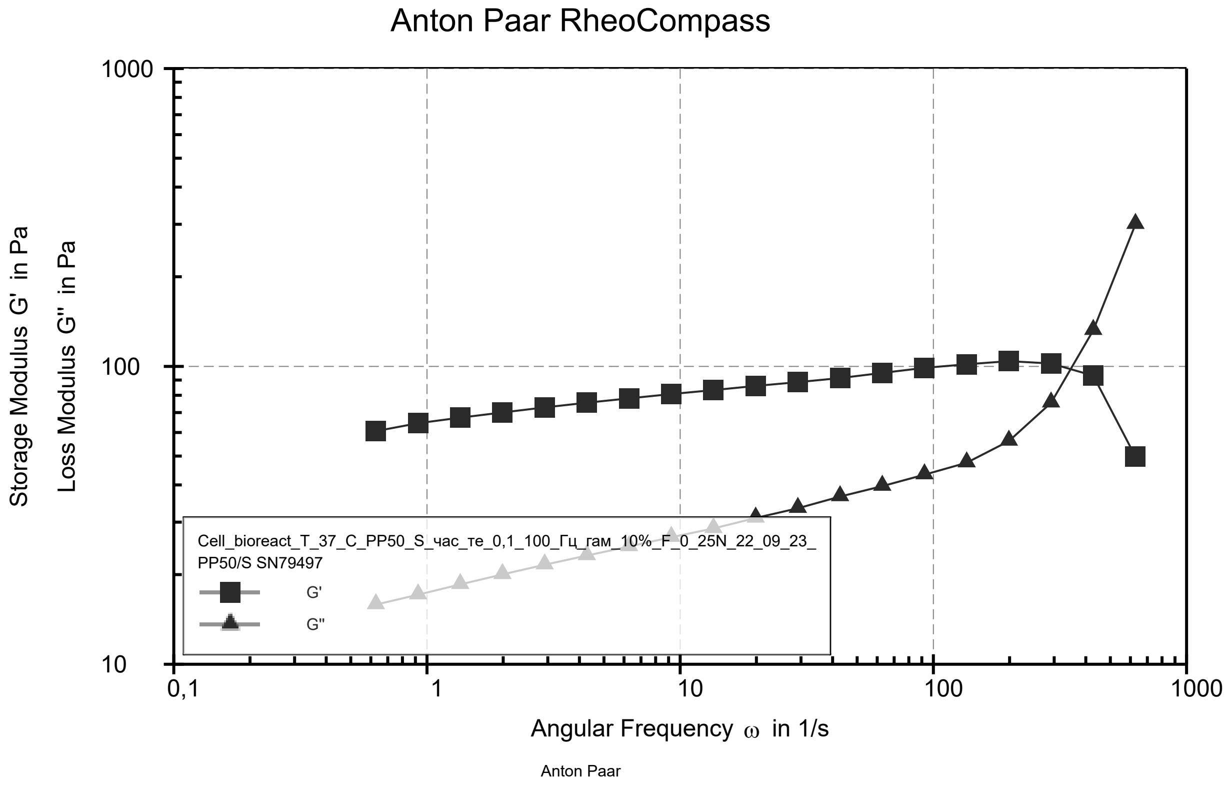

Cell_bioreact_T_37_C_PP50_S_час_те_0,1_100_Гц_гам_10%_F_0_25N_22_09_23_, Frequency sweep 1, Interval 1

Point N	Angul	Freque	Storag	Loss M	Loss F	Shear	Shear	Shear	Torque	Status	Averag	Tempe	Compl	Compl	Phase Shift Angle	Norma	Gap
№	ω	f	G'	G''	tan(δ)	γ	γ	τ	M	Stat	t_{avr}	T	|η*|	|G*|	δ	F_N	d
	[rad/s]	[Hz]	[Pa]	[Pa]	[1]	[%]	[1]	[Pa]	[mN·m		[s]	[°C]	[Pa·s]	[Pa]	[°]	[N]	[mm]
1	0,628	0,1	60,635	15,861	0,262	10,1	0,101	6,3052	0,2315 8	TruStra in™	75,19	37,00	99,75	62,675	14,66	0,42	0,210
2	0,922	0,147	64,472	17,122	0,266	10,1	0,101	6,7096	0,2464 4	TruStra in™	155,7	37,00	72,33	66,706	14,87	0,42	0,210
3	1,35	0,215	67,296	18,541	0,276	10	0,1	7,0119	0,2575	TruStra	240	37,00	51,566	69,803	15,40	0,41	0,210

Signature of operator: ____________________ Name: Date:

Company:
Street:
City:

Report

									4	in™							
4	1,99	0,316	70,045	20,028	0,286	10,1	0,101	7,3289	0,26918	TruStrain™	325,8	37,00	36,666	72,852	15,96	0,41	0,210
5	2,92	0,464	72,836	21,584	0,296	10	0,1	7,6269	0,28013	TruStrain™	412,8	37,00	26,048	75,966	16,51	0,41	0,210
6	4,28	0,681	75,523	23,169	0,307	10	0,1	7,9311	0,2913	TruStrain™	501,5	37,00	18,454	78,997	17,06	0,40	0,210
7	6,28	1	78,114	24,852	0,318	10,1	0,101	8,2464	0,30288	TruStrain™	590,5	37,00	13,046	81,972	17,65	0,40	0,210
8	9,22	1,47	80,759	26,696	0,331	10,1	0,101	8,5492	0,314	TruStrain™	680,1	37,00	9,2228	85,057	18,29	0,40	0,210
9	13,5	2,15	83,275	28,573	0,343	10	0,1	8,8438	0,32482	TruStrain™	769,3	37,00	6,5038	88,04	18,94	0,40	0,210
10	19,9	3,16	85,996	30,984	0,360	10,1	0,101	9,1972	0,33781	TruStrain™	858,6	37,00	4,6005	91,407	19,81	0,40	0,210
11	29,2	4,64	88,553	33,397	0,377	10	0,1	9,5027	0,34903	TruStrain™	947,9	37,00	3,2451	94,641	20,66	0,39	0,210
12	42,8	6,81	91,392	36,564	0,400	10,1	0,101	9,9008	0,36365	TruStrain™	1037	37,00	2,2995	98,435	21,81	0,39	0,210
13	62,8	10	95,1	39,623	0,417	10,1	0,101	10,365	0,38071	TruStrain™	1127	37,00	1,6397	103,02	22,62	0,39	0,210
14	92,2	14,7	98,89	43,303	0,438	10,1	0,101	10,855	0,39868	TruStrain™	1217	37,00	1,1706	107,96	23,65	0,39	0,210
15	135	21,5	101,65	47,488	0,467	10,1	0,101	11,289	0,41464	TruStrain™	1307	37,00	0,82884	112,2	25,04	0,39	0,210
16	199	31,6	104,15	56,144	0,539	10	0,1	11,885	0,43651	TruStrain™	1397	37,00	0,5955	118,32	28,33	0,39	0,210
17	292	46,4	102,29	75,46	0,738	10	0,1	12,77	0,46904	TruStrain™	1487	37,00	0,43584	127,11	36,42	0,39	0,210
18	428	68,1	93,087	132,2	1,420	10	0,1	16,232	0,5962	TruStrain™	1577	37,00	0,3777	161,68	54,85	0,39	0,210
19	628	100	49,759	300,02	6,030	10	0,1	30,534	1,1215	TruStrain™	1667	37,00	0,48402	304,12	80,58	0,39	0,210

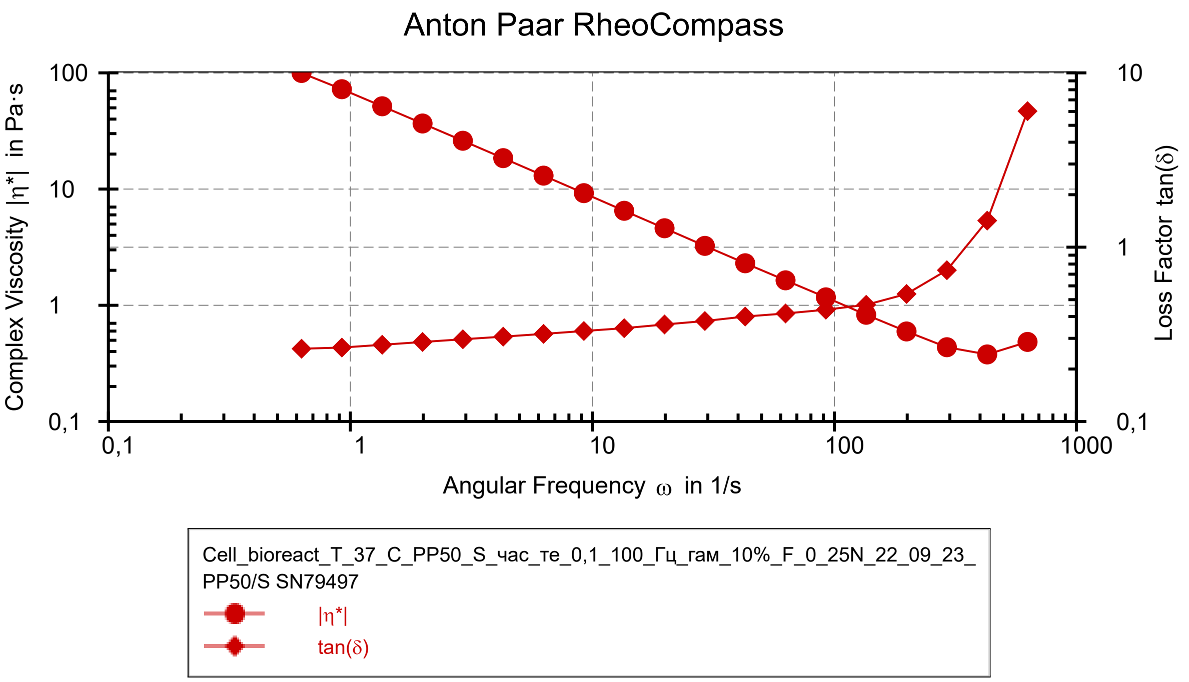

Anton Paar

Signature of operator: ______________________ Name: Date:

Company:
Street:
City:

Report

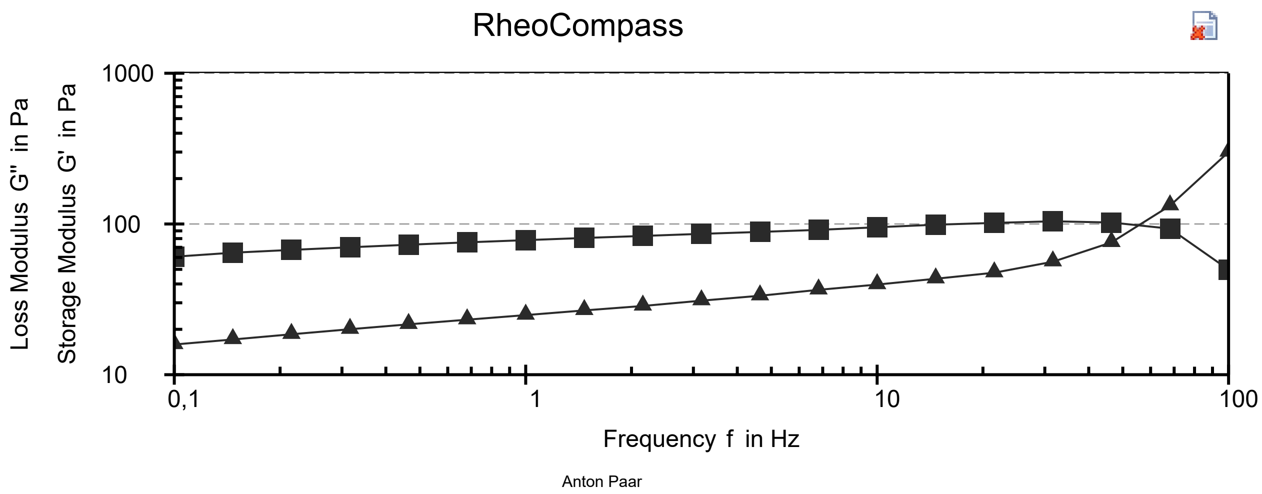

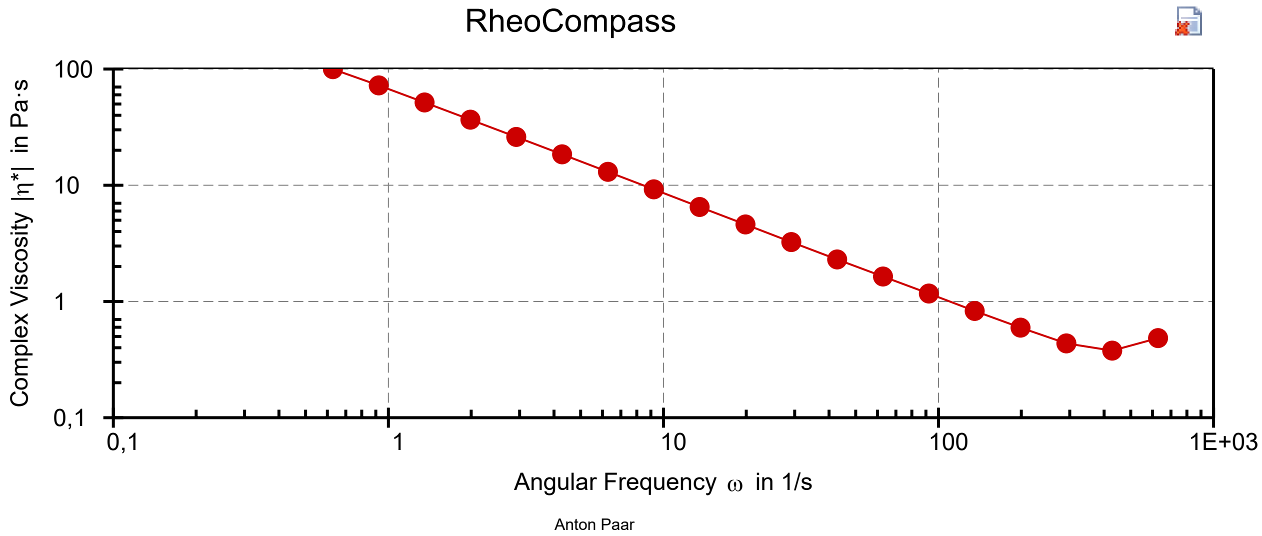

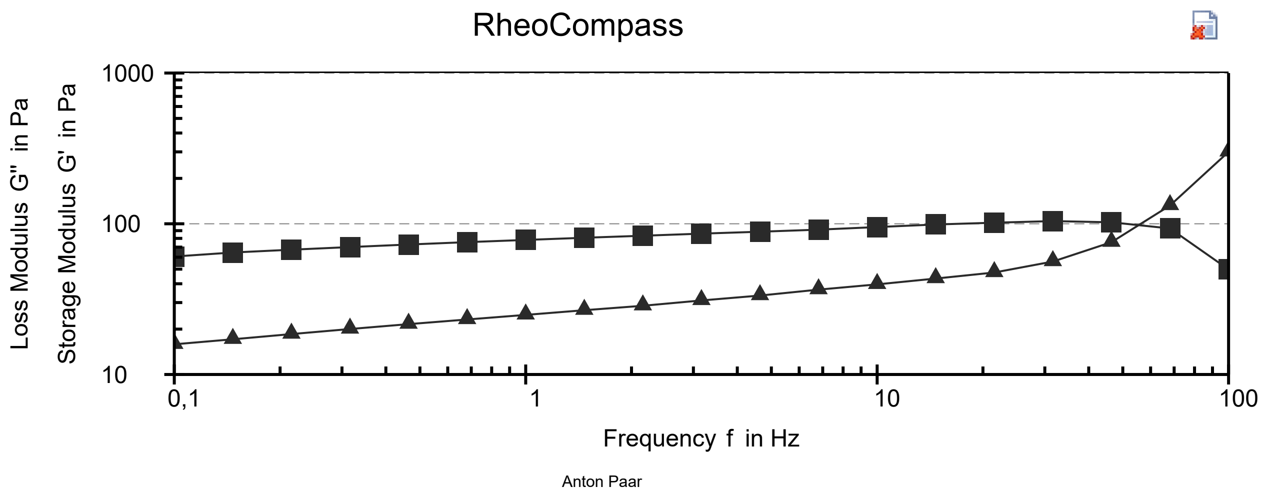

Text

Signature of operator: ______________________ Name: Date:
