# Supplementary material for: Structural and Viscoelastic Properties of Bacterial Cellulose Composites: Implications for Prosthetics
Source: Polymers (Basel). 2024 Nov 18;16(22):3200. doi: 10.3390/polym16223200 (PMC11597974; doi:10.3390/polym16223200)
Supplement: Supplementary file 1 [file polymers-16-03200-s001.zip › Cell_H_o┤_37_o│_PP50_S_oΘo╤oπ_oΣo╓_0,1_100_oñoΦ_o╘o╤o▐_10%_F_0_25N_08_08_23__15_17_42.pdf]

Company:
Street:
City:

Report

Test | Info Cell_H_T_37_C_PP50_S_час_те_0,1_100_Гц_гам_10%_F_0.25N_08_08_23_

Test created by operator: temp
Test creation date: 08.08.2023 12:38:25

Origin of project:

Rheometer: MCR 302 SN82961886
Measuring System: PP50/S SN79497

-

Sample | Info
Sample name:
Batch No.:

Description:

-

Result Data
Viscosity | 1st point:
Viscosity | last point:

Regression:
Interpolation:

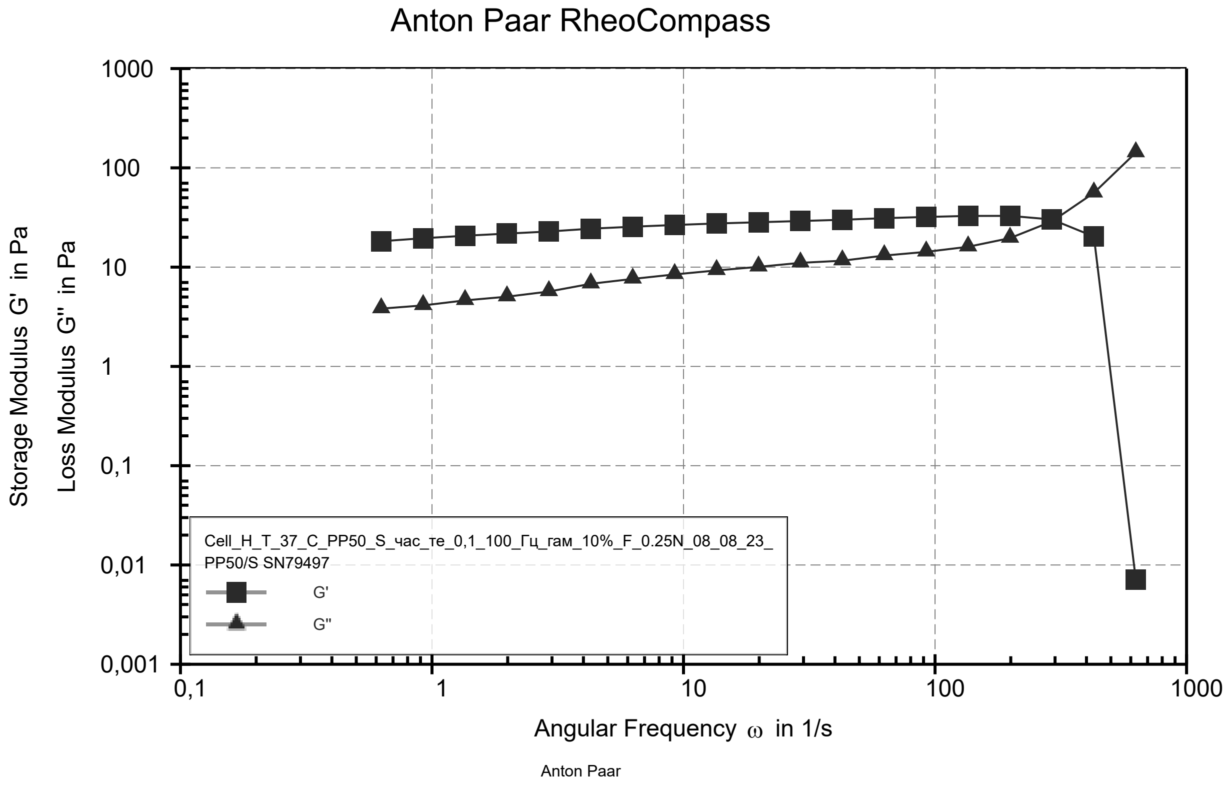

Cell_H_T_37_C_PP50_S_час_те_0,1_100_Гц_гам_10%_F_0.25N_08_08_23_, Frequency sweep 1, Interval 1

Point N	Angula	Freque	Storag	Loss M	Loss Fa	Shear S	Shear S	Shear S	Torque	Status	Averag	Tempe	Compl	Compl	Phase Shift Angle	Norma	Gap
№	ω	f	G'	G''	tan(δ)	γ	γ	τ	M	Stat	t_{avr}	T	|η*|	|G*|	δ	F_N	d
	[rad/s]	[Hz]	[Pa]	[Pa]	[1]	[%]	[1]	[Pa]	[mN·m		[s]	[°C]	[Pa·s]	[Pa]	[°]	[N]	[mm]
1	0,628	0,1	18,23	3,82	0,210	10	0,1	1,8715	0,0687 39	TruStra in™	75,19	37,00	29,644	18,626	11,83	0,04	0,105
2	0,922	0,147	19,55	4,1137	0,210	10,1	0,101	2,0097	0,0738 14	TruStra in™	155,7	37,00	21,663	19,978	11,88	0,04	0,105
3	1,35	0,215	20,712	4,6245	0,223	10	0,1	2,1304	0,0782	TruStra	240	37,00	15,677	21,222	12,59	0,04	0,105

Signature of operator: ______________________ Name: Date:

Report

									46	in™									
4	1,99	0,316	21,758	5,0369	0,232	10	0,1	2,2428	0,082377	TruStrain™	325,8	37,00	11,24	22,333	13,03			0,04	0,105
5	2,92	0,464	22,853	5,6775	0,248	10,1	0,101	2,3695	0,087031	TruStrain™	412,8	37,00	8,0744	23,548	13,95			0,03	0,105
6	4,28	0,681	24,397	6,7949	0,279	10,1	0,101	2,5459	0,09351	TruStrain™	501,5	37,00	5,9163	25,326	15,56			0,03	0,105
7	6,28	1	25,534	7,6073	0,298	10,1	0,101	2,6784	0,098376	TruStrain™	590,5	37,00	4,2404	26,643	16,59			0,03	0,105
8	9,22	1,47	26,58	8,4465	0,318	10,1	0,101	2,8059	0,10306	TruStrain™	679,7	37,00	3,0241	27,89	17,63			0,03	0,105
9	13,5	2,15	27,515	9,2677	0,337	10,1	0,101	2,9204	0,10727	TruStrain™	769	37,00	2,1448	29,034	18,61			0,03	0,105
10	19,9	3,16	28,326	10,064	0,355	10	0,1	3,0208	0,11095	TruStrain™	858,6	37,00	1,5129	30,06	19,56			0,03	0,105
11	29,2	4,64	29,146	11,042	0,379	10	0,1	3,1312	0,11501	TruStrain™	947,8	37,00	1,0687	31,168	20,75			0,03	0,105
12	42,8	6,81	29,991	11,607	0,387	10	0,1	3,23	0,11863	TruStrain™	1037	37,00	0,75124	32,158	21,16			0,03	0,105
13	62,8	10	31,127	13,078	0,420	10,1	0,101	3,3946	0,12468	TruStrain™	1127	37,00	0,53735	33,763	22,79			0,03	0,105
14	92,2	14,7	32,024	14,25	0,445	10,1	0,101	3,5249	0,12947	TruStrain™	1217	37,00	0,38007	35,052	23,99			0,03	0,105
15	135	21,5	32,796	16,038	0,489	10,1	0,101	3,6695	0,13478	TruStrain™	1307	37,00	0,26969	36,507	26,06			0,03	0,105
16	199	31,6	32,837	19,6	0,597	10,1	0,101	3,8447	0,14121	TruStrain™	1397	37,00	0,19247	38,242	30,83			0,03	0,105
17	292	46,4	30,189	28,873	0,956	10,1	0,101	4,1994	0,15424	TruStrain™	1487	37,00	0,14324	41,774	43,72			0,03	0,105
18	428	68,1	20,34	55,979	2,752	10,1	0,101	5,9914	0,22006	TruStrain™	1577	37,00	0,13914	59,56	70,03			0,03	0,105
19	628	100	0,0071167	142,33	20000,000	10,1	0,101	14,319	0,52593	ME-,taD,TruStrain™	1667	37,00	0,22653	142,33	90,00			0,03	0,105

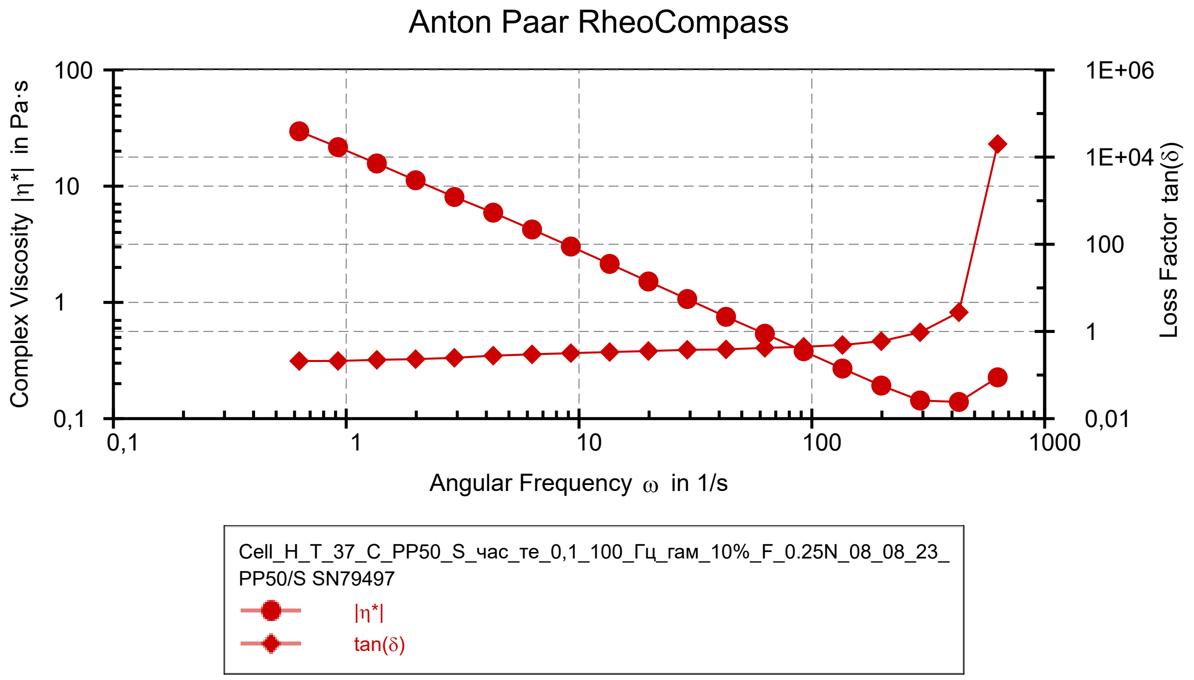

Anton Paar

Signature of operator: ____________________ Name: Date:

Company:
Street:
City:

Report

RheoCompass

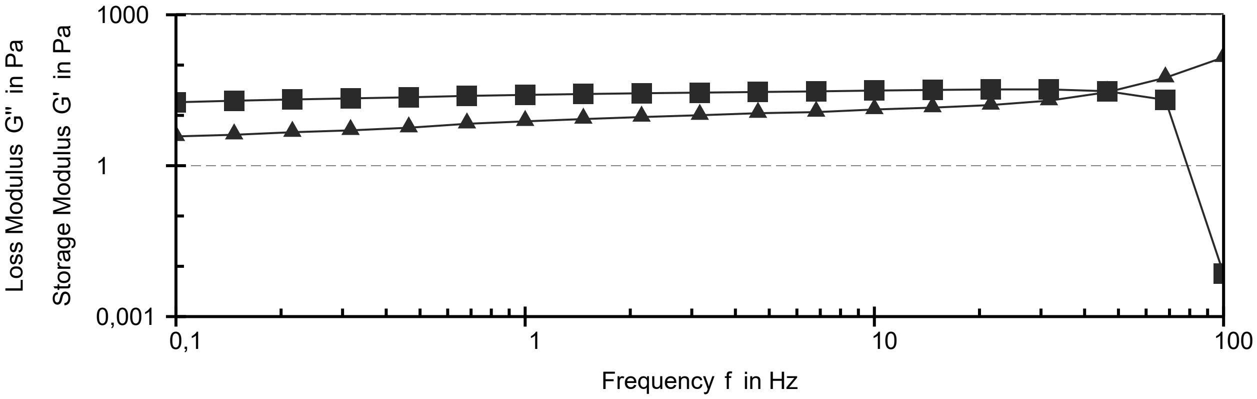

Anton Paar

RheoCompass

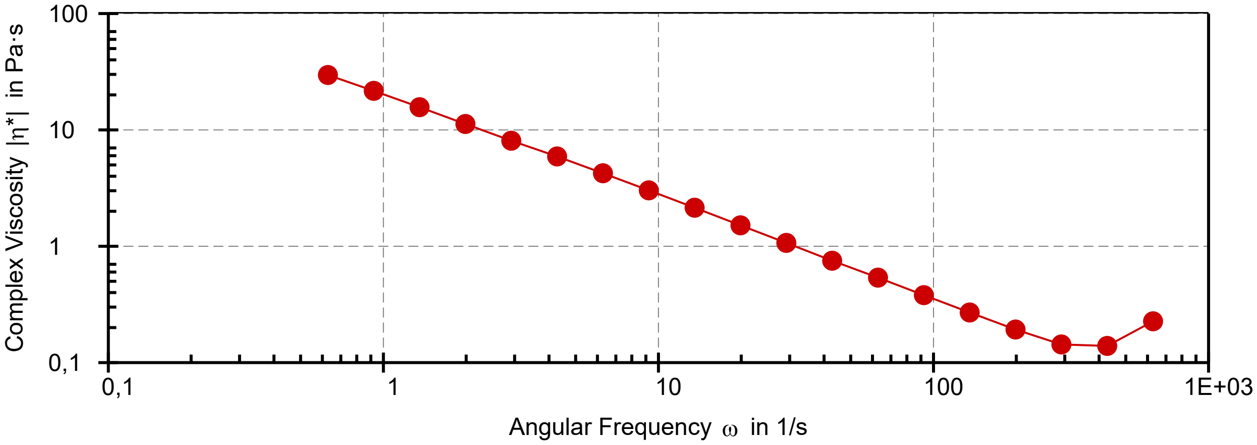

Anton Paar

RheoCompass

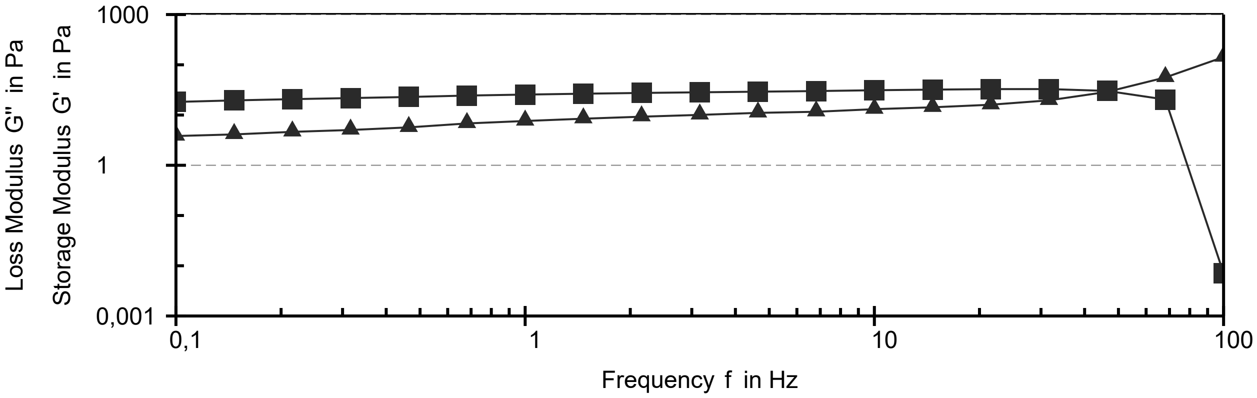

Anton Paar

Text

Signature of operator: ______________________ Name: Date:
