# Supplementary material for: Structural and Viscoelastic Properties of Bacterial Cellulose Composites: Implications for Prosthetics
Source: Polymers (Basel). 2024 Nov 18;16(22):3200. doi: 10.3390/polym16223200 (PMC11597974; doi:10.3390/polym16223200)
Supplement: Supplementary file 1 [file polymers-16-03200-s001.zip › Cell_Re_H_o┤_37_o│_PP50_S_oΘo╤oπ_oΣo╓_0,1_100_oñoΦ_o╘o╤o▐_10%_F_0_25N_09_08_23__11_55_43.pdf]

Company:
Street:
City:

Report

Test | Info

Cell_Re_H_T_37_C_PP50_S_час_те_0,1_100_Гц_гам_10%_F_0_25N_09_08_23_

Test created by operator:	temp
Test creation date:	09.08.2023 11:22:40
Origin of project:	
Rheometer:	MCR 302 SN82961886
Measuring System:	PP50/S SN79497

-

Sample | Info

Sample name:
Batch No.:

Description:

-

Result Data

Viscosity | 1st point:
Viscosity | last point:

Regression:
Interpolation:

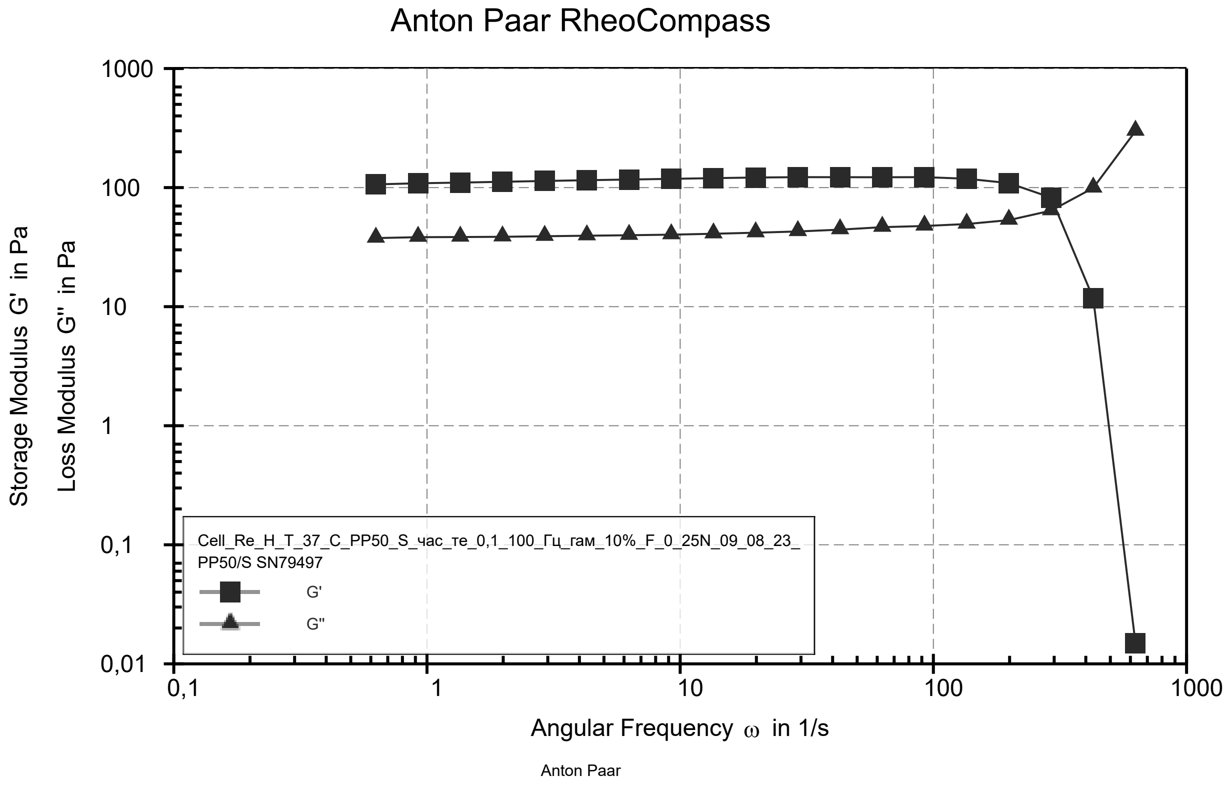

Cell_Re_H_T_37_C_PP50_S_час_те_0,1_100_Гц_гам_10%_F_0_25N_09_08_23_, Frequency sweep 1, Interval 1

Point N<br>№	Angul<br>ω<br>[rad/s]	Freque<br>f<br>[Hz]	Storag<br>G'<br>[Pa]	Loss M<br>G''<br>[Pa]	Loss Fa<br>tan(δ)<br>[1]	Shear :<br>γ<br>[%]	Shear :<br>γ<br>[1]	Shear :<br>τ<br>[Pa]	Torque<br>M<br>[mN·m	Status<br>Stat	Averag<br>t_{avr}<br>[s]	Tempe<br>T<br>[°C]	Compl<br>|η*| [Pa·s]	Compl<br>|G*| [Pa]	Phase Shift Angle<br>δ<br>[°]	Norma<br>F_N<br>[N]	Gap<br>d<br>[mm]
1	0,628	0,1	106,4	37,656	0,354	10,1	0,101	11,343	0,4166<br>3	TruStra<br>in™	75,24	37,00	179,63	112,87	19,49	0,06	0,126
2	0,922	0,147	108,67	38,319	0,353	10,1	0,101	11,582	0,4253<br>8	TruStra<br>in™	155,7	37,00	124,94	115,23	19,42	0,05	0,126
3	1,35	0,215	110,16	38,431	0,349	10	0,1	11,725	0,4306	TruStra	240	37,00	86,186	116,67	19,23	0,04	0,126

Signature of operator: ____________________________ Name: Date:

Report

									5	in™							
4	1,99	0,316	111,87	38,611	0,345	10,1	0,101	11,896	0,43692	TruStrain™	325,8	37,00	59,563	118,35	19,04	0,03	0,126
5	2,92	0,464	113,65	39,037	0,343	10,1	0,101	12,078	0,44362	TruStrain™	412,8	37,00	41,203	120,17	18,96	0,03	0,126
6	4,28	0,681	115,34	39,43	0,342	10,1	0,101	12,253	0,45003	TruStrain™	501,5	37,00	28,476	121,9	18,87	0,03	0,126
7	6,28	1	116,88	39,786	0,340	10	0,1	12,408	0,45574	TruStrain™	590,5	37,00	19,651	123,47	18,80	0,02	0,126
8	9,22	1,47	118,43	40,247	0,340	10,1	0,101	12,571	0,46172	TruStrain™	679,7	37,00	13,563	125,08	18,77	0,02	0,126
9	13,5	2,15	120,03	40,94	0,341	10	0,1	12,745	0,46811	TruStrain™	769	37,00	9,3689	126,82	18,83	0,02	0,126
10	19,9	3,16	121,46	41,795	0,344	10,1	0,101	12,911	0,47422	TruStrain™	858,5	37,00	6,465	128,45	18,99	0,02	0,126
11	29,2	4,64	122,37	42,79	0,350	10	0,1	13,028	0,4785	TruStrain™	947,7	37,00	4,4449	129,63	19,27	0,02	0,126
12	42,8	6,81	122,27	44,348	0,363	10,1	0,101	13,072	0,48014	TruStrain™	1038	37,00	3,0385	130,07	19,94	0,01	0,126
13	62,8	10	122,13	46,504	0,381	10,1	0,101	13,136	0,48247	TruStrain™	1127	37,00	2,0799	130,69	20,85	0,01	0,126
14	92,2	14,7	122,36	47,591	0,389	10,1	0,101	13,195	0,48464	TruStrain™	1217	37,00	1,4236	131,29	21,25	0,01	0,126
15	135	21,5	118,69	49,415	0,416	10	0,1	12,92	0,47454	TruStrain™	1307	37,00	0,94977	128,57	22,60	0,01	0,126
16	199	31,6	109,03	53,314	0,489	10,1	0,101	12,2	0,44808	TruStrain™	1397	37,00	0,61085	121,37	26,06	0,01	0,126
17	292	46,4	82,382	64,051	0,777	10	0,1	10,487	0,38517	TruStrain™	1487	37,00	0,35781	104,35	37,86	0,00	0,126
18	428	68,1	11,751	99,26	8,447	10,1	0,101	10,046	0,36897	TruStrain™	1577	37,00	0,2335	99,954	83,25	0,00	0,126
19	628	100	0,014895	297,89	20000,000	10	0,1	29,937	1,0996	ME-,taD,TruStrain™	1667	37,00	0,47411	297,89	90,00	0,01	0,126

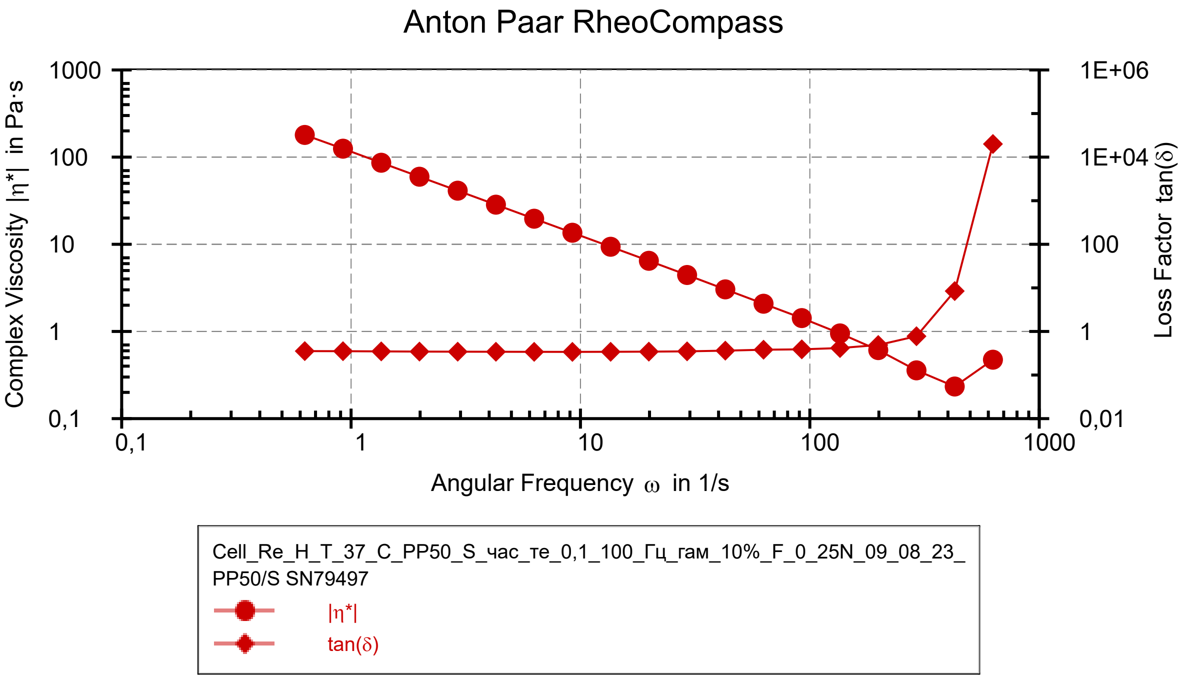

Anton Paar

Signature of operator: ________________________ Name: Date:

Company:
Street:
City:

Report

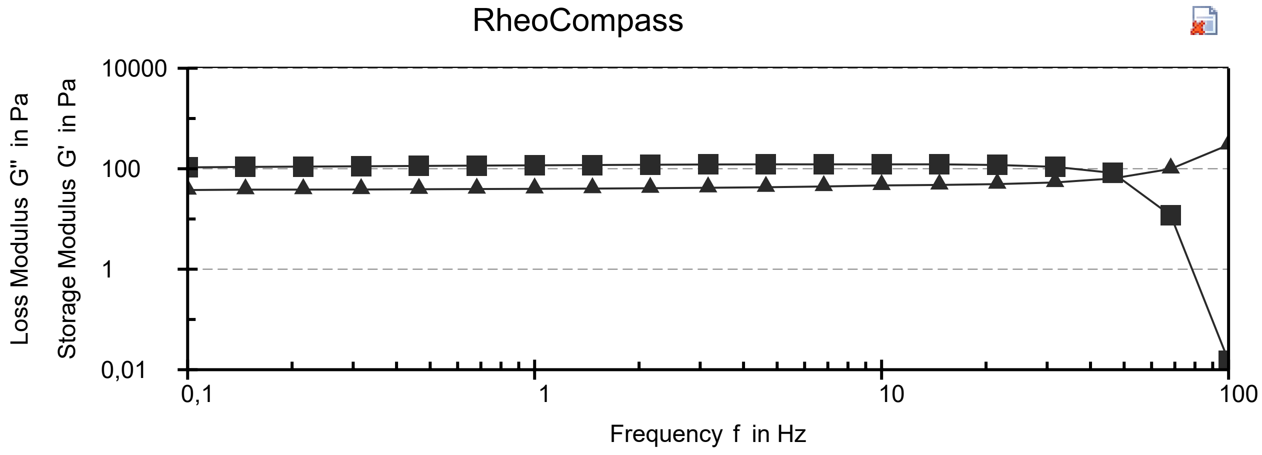

Anton Paar

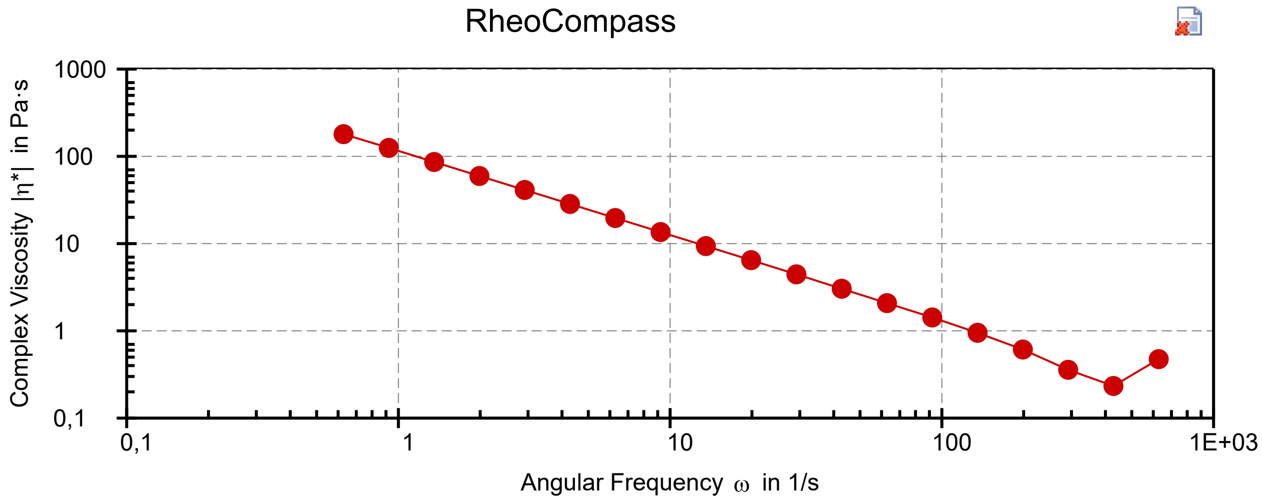

Anton Paar

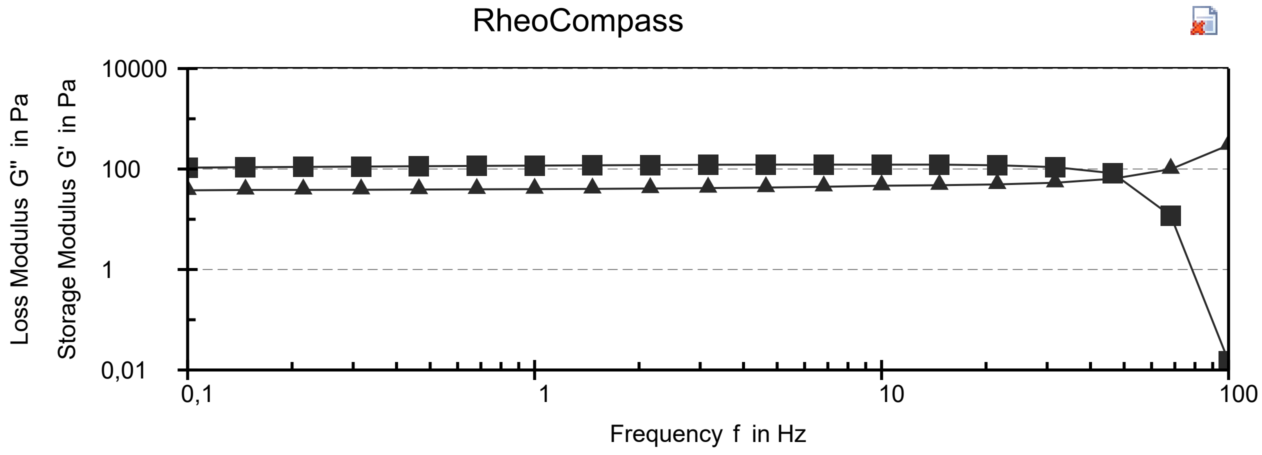

Anton Paar

Text

Signature of operator: ______________________ Name: Date:
