# Supplementary material for: Structural and Viscoelastic Properties of Bacterial Cellulose Composites: Implications for Prosthetics
Source: Polymers (Basel). 2024 Nov 18;16(22):3200. doi: 10.3390/polym16223200 (PMC11597974; doi:10.3390/polym16223200)
Supplement: Supplementary file 1 [file polymers-16-03200-s001.zip › Cell_static_o┤_37_o│_PP50_S_oΘo╤oπ_oΣo╓_0,1_100_oñoΦ_o╘o╤o▐_10%_F_0_25N_22_09_23__13_01_59.pdf]

Company:
Street:
City:

Report

<u>Test | Info</u> Cell_static_T_37_C_PP50_S_час_те_0,1_100_Гц_гам_10%_F_0_25N_22_09_23_

Test created by operator: temp

Test creation date: 22.09.2023 12:31:16

Origin of project:

Rheometer: MCR 302 SN82961886

Measuring System: PP50/S SN79497

-

<u>Sample | Info</u>

Sample name:

Batch No.:

Description:

-

Result Data

Viscosity | 1st point:

Viscosity | last point:

Regression:

Interpolation:

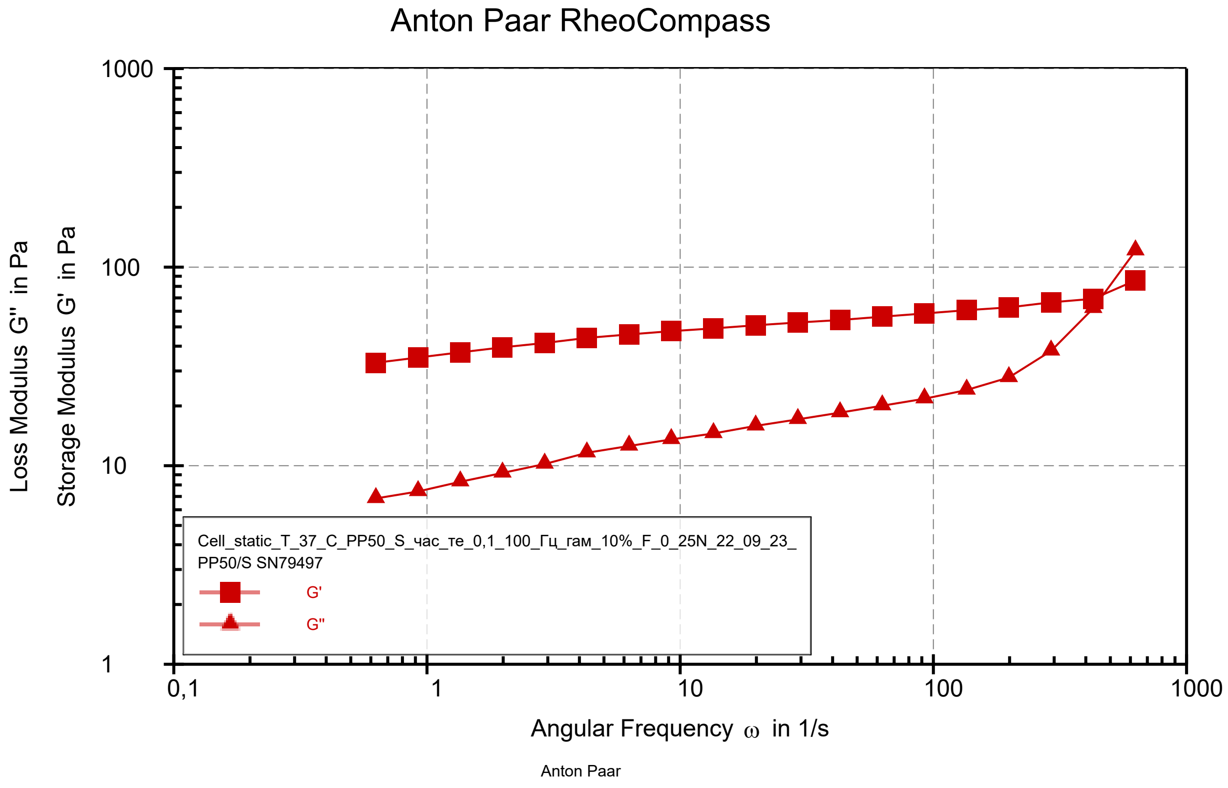

Cell_static_T_37_C_PP50_S_час_те_0,1_100_Гц_гам_10%_F_0_25N_22_09_23_, Frequency sweep 1, Interval 1

Point Nº	Angula ω [rad/s]	Freque f [Hz]	Storag G' [Pa]	Loss M G'' [Pa]	Loss Fa tan(δ) [1]	Shear ! γ [%]	Shear ! γ [1]	Shear ! τ [Pa]	Torque M [mN·m	Status Stat	Averag t_{avr} [s]	Tempe T [°C]	Compl |η*| [Pa·s]	Compl |G*| [Pa]	Phase Shift Angle δ [°]	Norma F_N [N]	Gap d [mm]
1	0,628	0,1	32,991	6,8398	0,207	10,1	0,101	3,3946	0,1246<br>8	TruStra<br>in™	75,1	37,00	53,623	33,693	11,71	0,19	0,098
2	0,922	0,147	35,057	7,4169	0,212	10,1	0,101	3,6054	0,1324<br>2	TruStra<br>in™	155,7	37,00	38,854	35,833	11,95	0,19	0,098
3	1,35	0,215	37,228	8,2995	0,223	10	0,1	3,8223	0,1403	TruStra	240	37,00	28,177	38,142	12,57	0,18	0,098

Signature of operator: ____________________ Name: Date:

Company:
Street:
City:

Report

									9	in™								
4	1,99	0,316	39,439	9,1938	0,233	10	0,1	4,0586	0,14907	TruStrain™	325,8	37,00	20,382	40,496	13,12		0,18	0,098
5	2,92	0,464	41,491	10,196	0,246	10,1	0,101	4,3039	0,15808	TruStrain™	412,8	37,00	14,65	42,725	13,81		0,18	0,098
6	4,28	0,681	44,009	11,614	0,264	10	0,1	4,5698	0,16784	TruStrain™	501,5	37,00	10,633	45,516	14,78		0,18	0,098
7	6,28	1	45,823	12,569	0,274	10	0,1	4,775	0,17538	TruStrain™	590,5	37,00	7,5624	47,516	15,34		0,17	0,098
8	9,22	1,47	47,587	13,561	0,285	10	0,1	4,9602	0,18218	TruStrain™	679,7	37,00	5,3654	49,482	15,91		0,18	0,098
9	13,5	2,15	49,137	14,531	0,296	10	0,1	5,1389	0,18875	TruStrain™	769	37,00	3,7853	51,241	16,47		0,17	0,098
10	19,9	3,16	50,949	15,866	0,311	10,1	0,101	5,3744	0,1974	TruStrain™	858,6	37,00	2,6857	53,363	17,30		0,17	0,098
11	29,2	4,64	52,62	17,113	0,325	10	0,1	5,5495	0,20383	TruStrain™	947,9	37,00	1,8973	55,333	18,02		0,17	0,098
12	42,8	6,81	54,163	18,501	0,342	10,1	0,101	5,7633	0,21168	TruStrain™	1038	37,00	1,3371	57,236	18,86		0,17	0,098
13	62,8	10	56,3	20,051	0,356	10	0,1	6,0046	0,22054	TruStrain™	1127	37,00	0,95117	59,764	19,60		0,17	0,098
14	92,2	14,7	58,435	21,735	0,372	10	0,1	6,2627	0,23002	TruStrain™	1217	37,00	0,67603	62,347	20,40		0,17	0,098
15	135	21,5	60,694	24,054	0,396	10,1	0,101	6,5789	0,24164	TruStrain™	1307	37,00	0,48229	65,286	21,62		0,17	0,098
16	199	31,6	62,687	27,815	0,444	10,1	0,101	6,9115	0,25385	TruStrain™	1397	37,00	0,34516	68,581	23,93		0,17	0,098
17	292	46,4	66,493	37,843	0,569	10	0,1	7,6727	0,28181	TruStrain™	1487	37,00	0,26234	76,507	29,65		0,17	0,098
18	428	68,1	69,264	61,995	0,895	10,1	0,101	9,3636	0,34392	TruStrain™	1577	37,00	0,21715	92,957	41,83		0,17	0,098
19	628	100	85,695	120,9	1,411	10,1	0,101	14,93	0,54835	TruStrain™	1667	37,00	0,23585	148,19	54,67		0,17	0,098

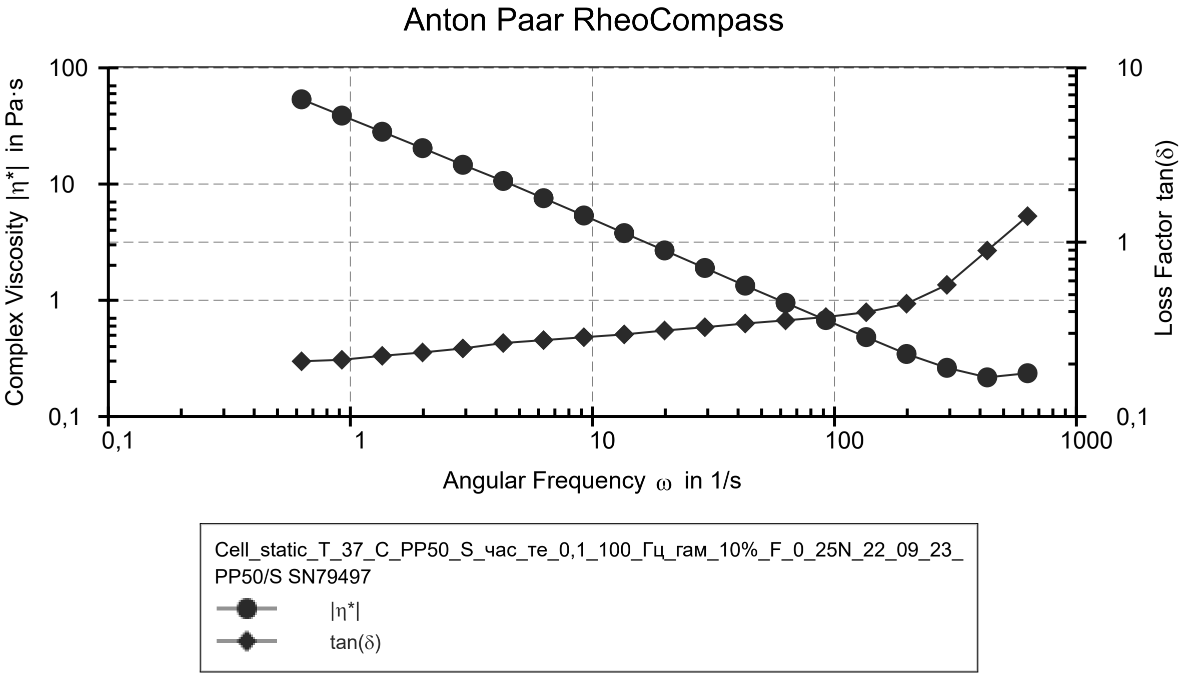

Anton Paar

Signature of operator: ______________________ Name: Date:

Company:
Street:
City:

Report

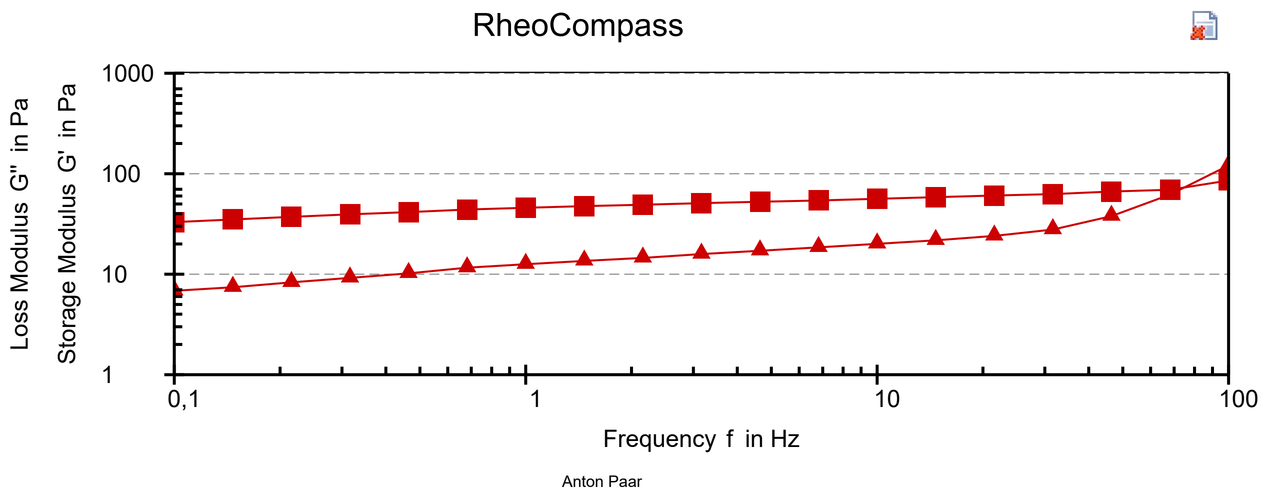

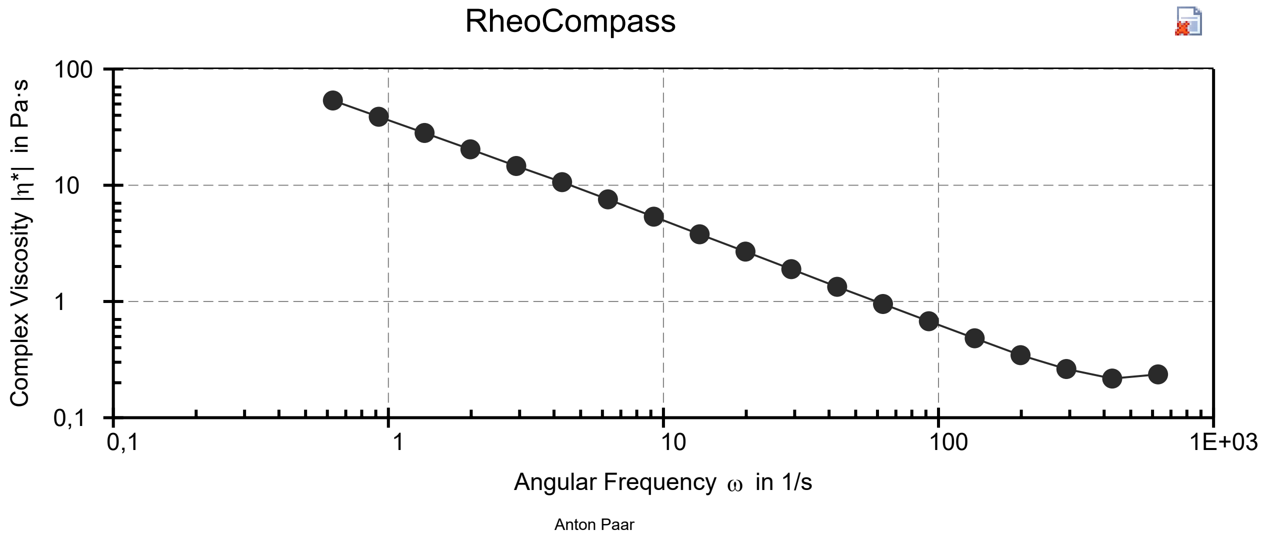

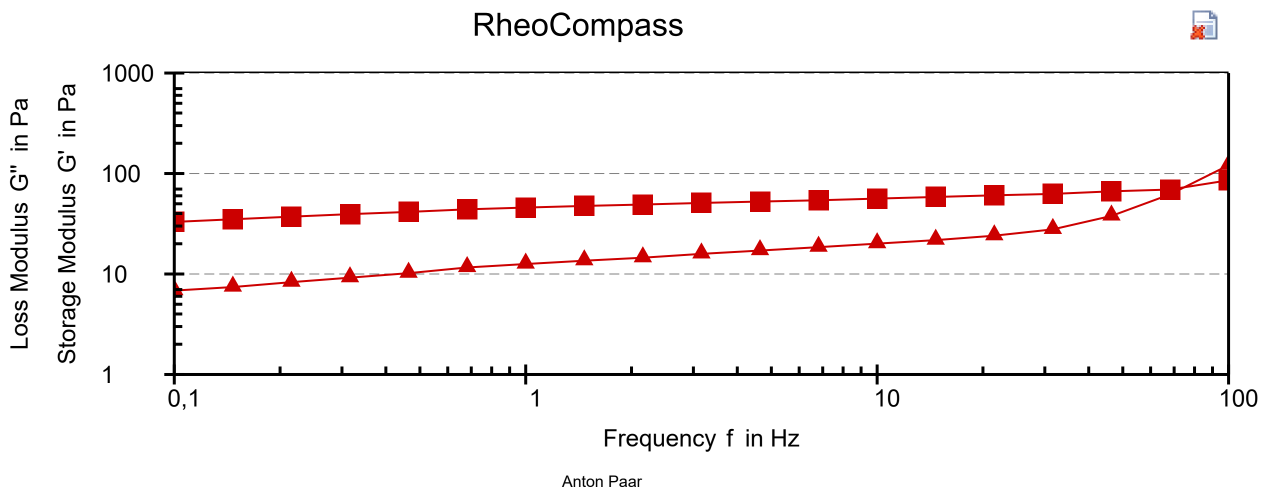

Text

Signature of operator: ________________________ Name: Date:
