# Supplementary material for: Structural and Viscoelastic Properties of Bacterial Cellulose Composites: Implications for Prosthetics
Source: Polymers (Basel). 2024 Nov 18;16(22):3200. doi: 10.3390/polym16223200 (PMC11597974; doi:10.3390/polym16223200)
Supplement: Supplementary file 1 [file polymers-16-03200-s001.zip › Cell_S_o┤_37_o│_PP50_S_oΘo╤oπ_oΣo╓_0,1_100_oñoΦ_o╘o╤o▐_10%_F_0_25N_08_08_23__16_33_25.pdf]

Company:
Street:
City:

# Report

## Test | Info

Cell_S_T_37_C_PP50_S_час_те_0,1_100_Гц_гам_10%_F_0_25N_08_08_23_

Test created by operator: temp

Test creation date: 08.08.2023 15:59:36

Origin of project:

Rheometer: MCR 302 SN82961886

Measuring System: PP50/S SN79497

-

## Sample | Info

Sample name:
Batch No.:

Description:

-

## Result Data

Viscosity | 1st point:
Viscosity | last point:

Regression:
Interpolation:

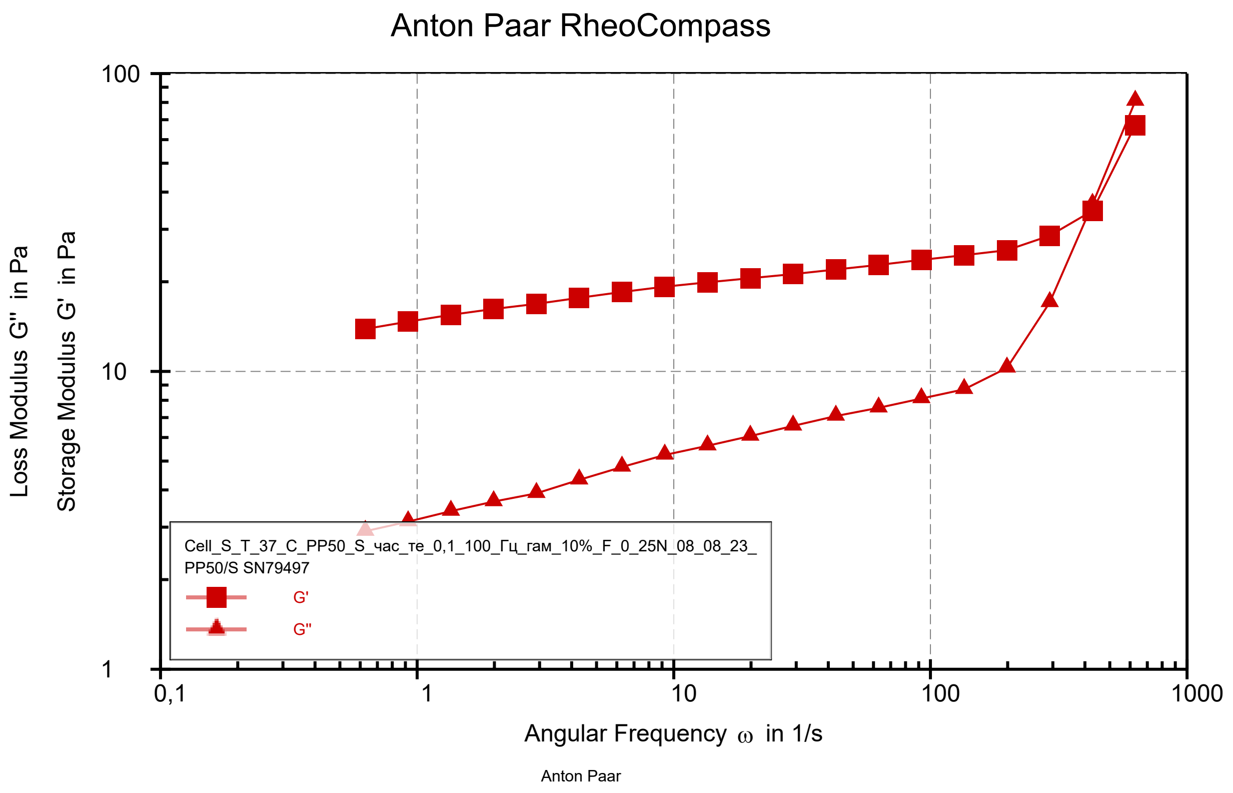


Cell_S_T_37_C_PP50_S_час_те_0,1_100_Гц_гам_10%_F_0_25N_08_08_23_, Frequency sweep 1, Interval 1

| Point N | Angula | Freque | Storag | Loss M | Loss Fa | Shear S | Shear S | Shear S | Torque | Status | Averag | Tempe | Compl | Compl | Phase Shift Angle | Norma | Gap |
|---|---|---|---|---|---|---|---|---|---|---|---|---|---|---|---|---|---|
| № | ω | f | G' | G'' | tan(δ) | γ | γ | τ | M | Stat | $t_{avr}$ | T | \|η*\| | \|G*\| | δ | $F_N$ | d |
| | [rad/s] | [Hz] | [Pa] | [Pa] | [1] | [%] | [1] | [Pa] | [mN·m | | [s] | [°C] | [Pa·s] | [Pa] | [°] | [N] | [mm] |
| 1 | 0,628 | 0,1 | 13,883 | 2,9076 | 0,209 | 10,1 | 0,101 | 1,4282 | 0,052458 | TruStrain™ | 75,13 | 37,00 | 22,575 | 14,184 | 11,83 | -0,14 | 0,076 |
| 2 | 0,922 | 0,147 | 14,694 | 3,1308 | 0,213 | 10,1 | 0,101 | 1,5121 | 0,055538 | TruStrain™ | 155,7 | 37,00 | 16,29 | 15,024 | 12,03 | -0,14 | 0,076 |
| 3 | 1,35 | 0,215 | 15,488 | 3,3955 | 0,219 | 10 | 0,1 | 1,5916 | 0,0584 | TruStra | 240 | 37,00 | 11,713 | 15,856 | 12,37 | -0,14 | 0,076 |

Signature of operator: ______________________ Name: Date:

Company:
Street:
City:

# Report

| | | | | | | | | | | | | | | | | | | |
|---|---|---|---|---|---|---|---|---|---|---|---|---|---|---|---|---|---|---|
| | | | | | | | | | 58 | in™ | | | | | | | | |
| 4 | 1,99 | 0,316 | 16,205 | 3,656 | 0,226 | 10 | 0,1 | 1,6673 | 0,061238 | TruStrain™ | 325,8 | 37,00 | 8,3607 | 16,612 | 12,71 | | -0,14 | 0,076 |
| 5 | 2,92 | 0,464 | 16,855 | 3,8964 | 0,231 | 10 | 0,1 | 1,7357 | 0,06375 | TruStrain™ | 412,8 | 37,00 | 5,9318 | 17,3 | 13,02 | | -0,15 | 0,076 |
| 6 | 4,28 | 0,681 | 17,665 | 4,3245 | 0,245 | 10 | 0,1 | 1,8255 | 0,067048 | TruStrain™ | 501,5 | 37,00 | 4,2485 | 18,187 | 13,76 | | -0,15 | 0,076 |
| 7 | 6,28 | 1 | 18,479 | 4,7761 | 0,258 | 10,1 | 0,101 | 1,919 | 0,070482 | TruStrain™ | 590,5 | 37,00 | 3,0376 | 19,086 | 14,49 | | -0,15 | 0,076 |
| 8 | 9,22 | 1,47 | 19,252 | 5,2415 | 0,272 | 10 | 0,1 | 2,0051 | 0,073646 | TruStrain™ | 679,7 | 37,00 | 2,1635 | 19,953 | 15,23 | | -0,15 | 0,076 |
| 9 | 13,5 | 2,15 | 19,911 | 5,628 | 0,283 | 10,1 | 0,101 | 2,083 | 0,076508 | TruStrain™ | 769 | 37,00 | 1,5286 | 20,692 | 15,78 | | -0,15 | 0,076 |
| 10 | 19,9 | 3,16 | 20,553 | 6,0662 | 0,295 | 10 | 0,1 | 2,1529 | 0,079075 | TruStrain™ | 858,3 | 37,00 | 1,0786 | 21,43 | 16,44 | | -0,15 | 0,076 |
| 11 | 29,2 | 4,64 | 21,215 | 6,5611 | 0,309 | 10 | 0,1 | 2,2304 | 0,081922 | TruStrain™ | 947,9 | 37,00 | 0,76144 | 22,207 | 17,18 | | -0,14 | 0,076 |
| 12 | 42,8 | 6,81 | 21,986 | 7,0816 | 0,322 | 10 | 0,1 | 2,3191 | 0,085177 | TruStrain™ | 1037 | 37,00 | 0,53959 | 23,098 | 17,85 | | -0,14 | 0,076 |
| 13 | 62,8 | 10 | 22,782 | 7,5472 | 0,331 | 10,1 | 0,101 | 2,4128 | 0,088621 | TruStrain™ | 1127 | 37,00 | 0,38197 | 24 | 18,33 | | -0,15 | 0,076 |
| 14 | 92,2 | 14,7 | 23,681 | 8,1089 | 0,342 | 10,1 | 0,101 | 2,5196 | 0,092543 | TruStrain™ | 1217 | 37,00 | 0,27141 | 25,031 | 18,90 | | -0,15 | 0,076 |
| 15 | 135 | 21,5 | 24,561 | 8,706 | 0,354 | 10 | 0,1 | 2,6143 | 0,096021 | TruStrain™ | 1307 | 37,00 | 0,1925 | 26,059 | 19,52 | | -0,15 | 0,076 |
| 16 | 199 | 31,6 | 25,526 | 10,272 | 0,402 | 10,1 | 0,101 | 2,7658 | 0,10158 | TruStrain™ | 1397 | 37,00 | 0,13848 | 27,515 | 21,92 | | -0,15 | 0,076 |
| 17 | 292 | 46,4 | 28,555 | 17,04 | 0,597 | 10,1 | 0,101 | 3,3427 | 0,12277 | TruStrain™ | 1487 | 37,00 | 0,11402 | 33,253 | 30,83 | | -0,15 | 0,076 |
| 18 | 428 | 68,1 | 34,633 | 36,423 | 1,052 | 10,1 | 0,101 | 5,0526 | 0,18558 | TruStrain™ | 1577 | 37,00 | 0,11741 | 50,26 | 46,44 | | -0,15 | 0,076 |
| 19 | 628 | 100 | 67,067 | 80,796 | 1,205 | 10,1 | 0,101 | 10,571 | 0,38827 | TruStrain™ | 1667 | 37,00 | 0,16712 | 105 | 50,30 | | -0,14 | 0,076 |

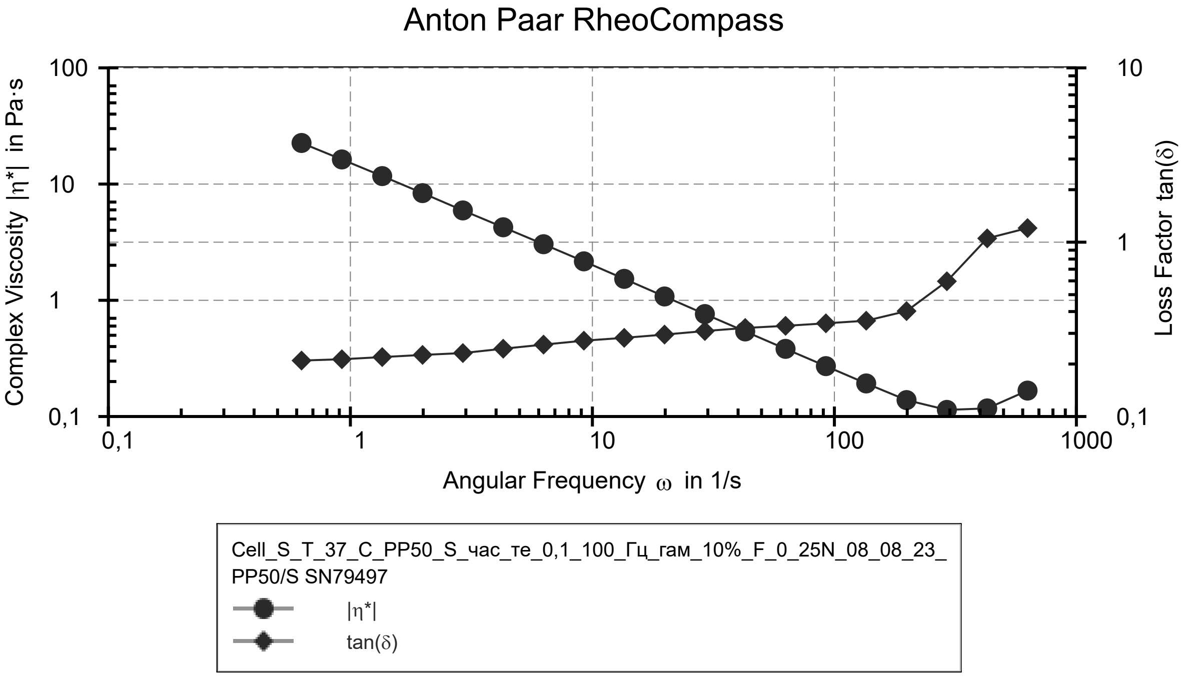


Anton Paar

Signature of operator: ____________________ Name: Date:

Company:
Street:
City:

# Report

RheoCompass

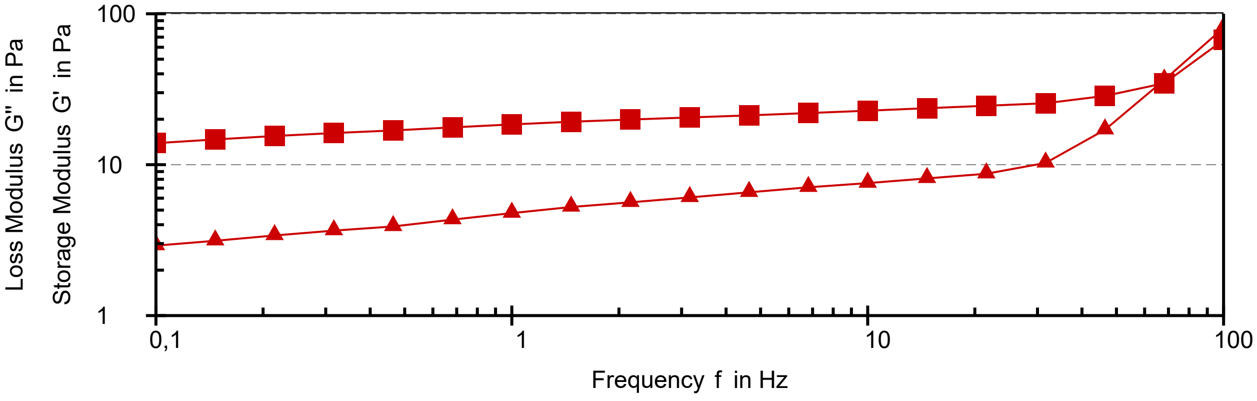


Anton Paar

RheoCompass

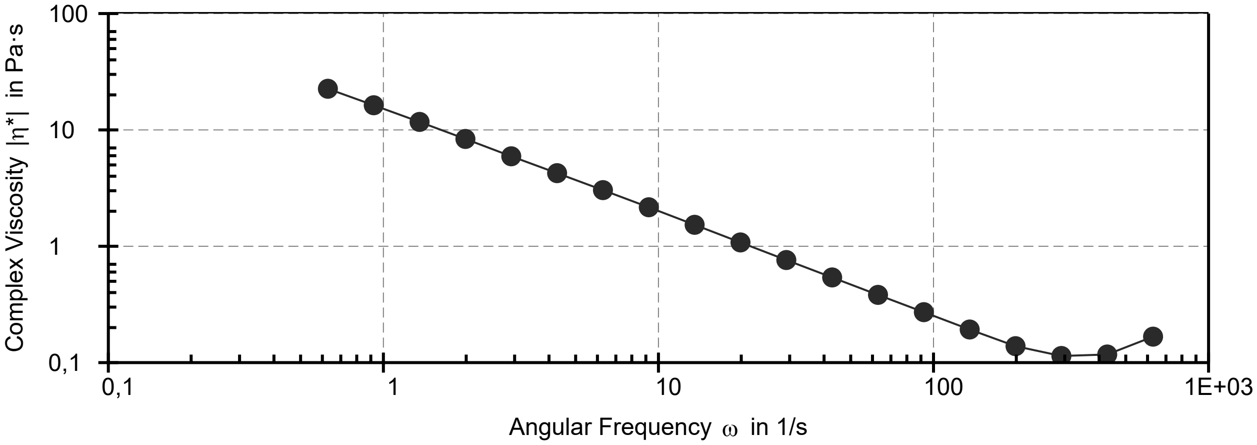


Anton Paar

RheoCompass

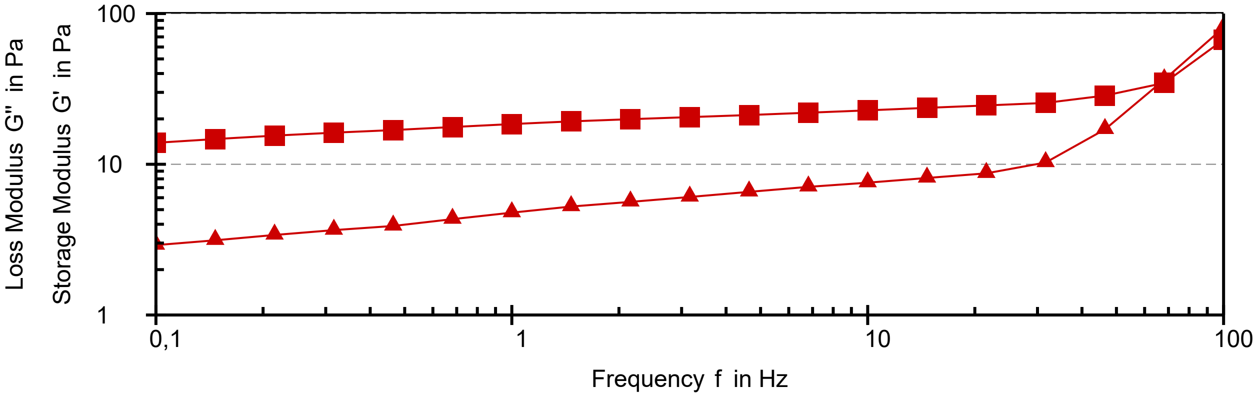


Anton Paar

Text

Signature of operator: ________________________ Name: Date:
