# Supplementary material for: Structural and Viscoelastic Properties of Bacterial Cellulose Composites: Implications for Prosthetics
Source: Polymers (Basel). 2024 Nov 18;16(22):3200. doi: 10.3390/polym16223200 (PMC11597974; doi:10.3390/polym16223200)
Supplement: Supplementary file 1 [file polymers-16-03200-s001.zip › Cel_bioreact_o┤_37_o│_PP50_S_F_0_25N_Amp_te _o╘o╤o▐_0_01_20%_f_1_Hz_22_09_23_11_37_49.pdf]

Company:
Street:
City:

Report

Test | Info Cel_bioreact_T_37_C_PP50_S_F_0_25N_Amp_te _гам_0_01_20%_f_1_Hz_22_09_23_

Test created by operator: temp

Test creation date: 22.09.2023 11:30:22

Origin of project:

Rheometer: MCR 302 SN82961886

Measuring System: PP50/S SN79497

-

Sample | Info

Sample name:

Batch No.:

Description:

-

Result Data

LVE Limit:

LVE Proposal:

Flow Point TAU_y: tau = 0,0309 Pa; gamma = 0,01664 %; G' = 168,5 Pa
(if applicable)

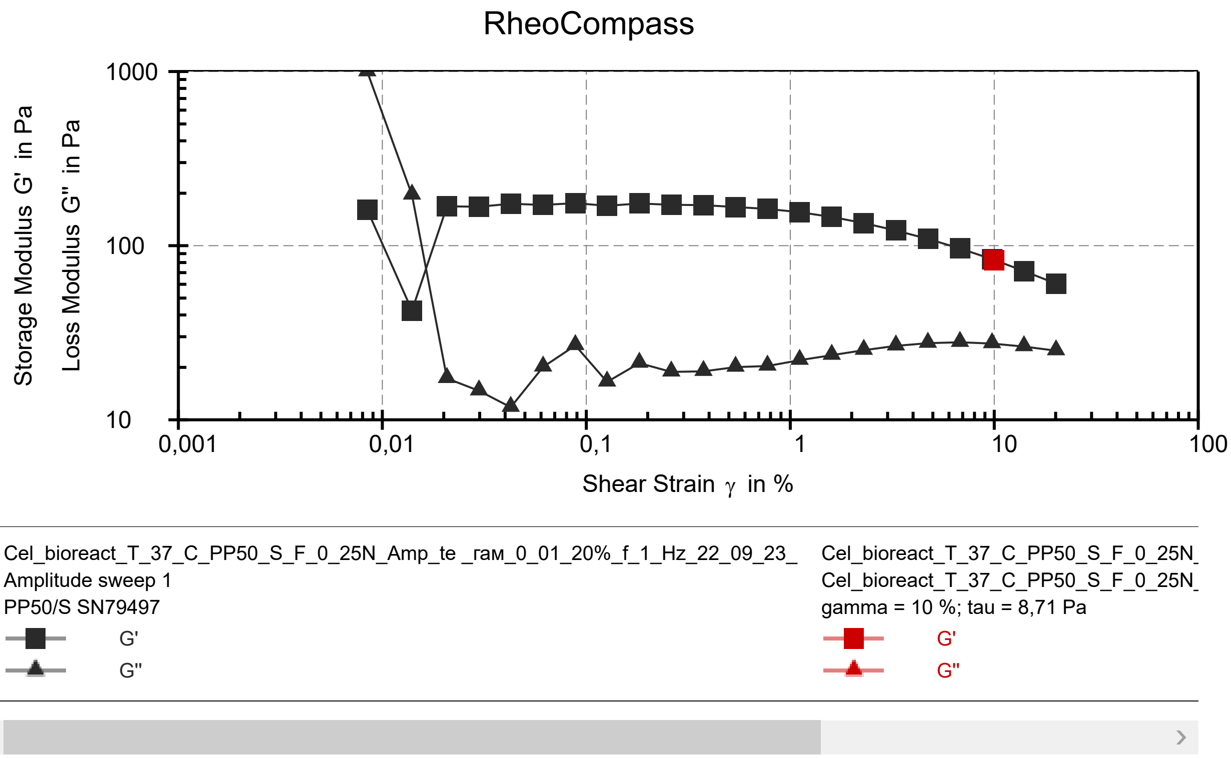

Anton Paar

Signature of operator: ______________________ Name: Date:

Company:
Street:
City:

Report

RheoCompass

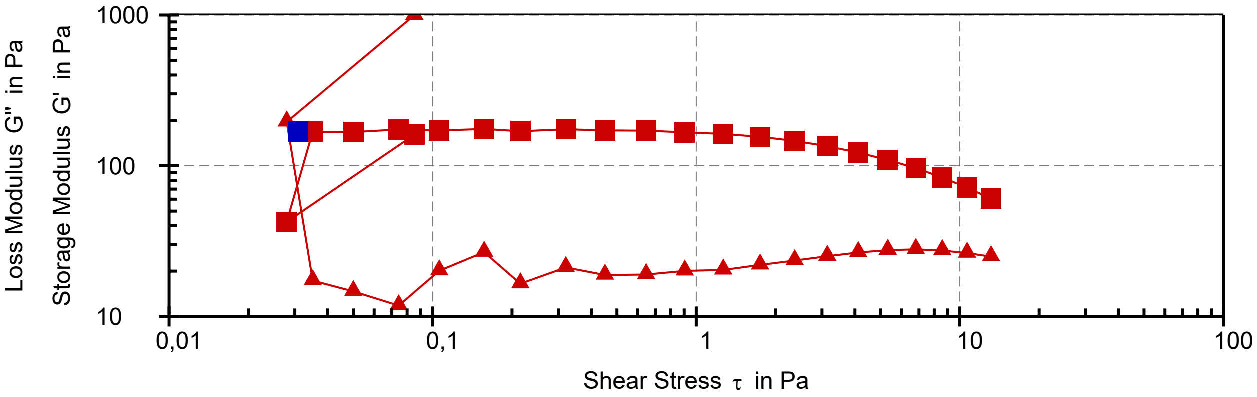

Anton Paar

Cel_bioreact_T_37_C_PP50_S_F_0_25N_Amp_te _raм_0_01_20%_f_1_Hz_22_09_23_, Amplitude sweep 1, Interval 1

Point No. Nº	Shear Str γ [1]	Shear Str γ [%]	Shear Str τ [Pa]	Storage M G' [Pa]	Loss Moc G'' [Pa]	Loss Fact tan(δ) [1]	Torque M [μN·m]	Status Stat	Time t [s]	Frequenc f [Hz]	Angular F ω [rad/s]	Time of D t_{abs}	Phase Sh δ [°]	Complex |G*| [Pa]	Temperat T [°C]	Gap d [mm]	Normal F F_N [N]
1	8,43E-05	0,00843	0,085225	160,69	997,6	6,208	3,1302	MV-,WMa,TruStrain™	28,39	1	6,28	11:31:02	80,85	1010,5	37,02	0,210	0,56
2	0,00014	0,014	0,027952	42,399	195,43	4,609	1,0266	WMa,TruStrain™	56,77	1	6,28	11:31:30	77,76	199,98	37,02	0,210	0,54
3	0,000207	0,0207	0,035073	168,24	17,285	0,103	1,2882	TruStrain™	78,47	1	6,28	11:31:52	5,87	169,13	37,02	0,210	0,53
4	0,000298	0,0298	0,050006	167,27	14,67	0,088	1,8367	TruStrain™	97,07	1	6,28	11:32:11	5,01	167,91	37,02	0,210	0,52
5	0,000426	0,0426	0,074347	174,06	11,807	0,068	2,7307	TruStrain™	114,2	1	6,28	11:32:28	3,88	174,46	37,01	0,210	0,51
6	0,000614	0,0614	0,10593	171,32	20,112	0,117	3,8908	TruStrain™	131	1	6,28	11:32:44	6,70	172,5	37,01	0,210	0,51
7	0,000884	0,0884	0,15681	175,45	26,72	0,152	5,7596	TruStrain™	148,7	1	6,28	11:33:02	8,66	177,48	37,01	0,210	0,50
8	0,00126	0,126	0,21516	169,4	16,494	0,097	7,9028	TruStrain™	166,4	1	6,28	11:33:20	5,56	170,2	37,01	0,210	0,50
9	0,00182	0,182	0,32005	174,64	21,155	0,121	11,755	TruStrain™	183,9	1	6,28	11:33:37	6,91	175,91	37,01	0,210	0,50
10	0,00261	0,261	0,45059	171,66	18,836	0,110	16,55	TruStrain™	201,4	1	6,28	11:33:55	6,26	172,69	37,00	0,210	0,49
11	0,00375	0,375	0,64554	170,9	18,98	0,111	23,71	TruStrain™	219,4	1	6,28	11:34:13	6,34	171,95	37,01	0,210	0,49
12	0,00539	0,539	0,90473	166,65	20,068	0,120	33,23	TruStrain™	237,2	1	6,28	11:34:31	6,87	167,86	37,00	0,210	0,48
13	0,00773	0,773	1,2664	162,61	20,359	0,125	46,515	TruStrain™	254,5	1	6,28	11:34:48	7,14	163,88	37,00	0,210	0,48
14	0,0111	1,11	1,7468	155,63	21,989	0,141	64,157	TruStrain™	271,8	1	6,28	11:35:05	8,04	157,18	37,00	0,210	0,47
15	0,016	1,6	2,3641	146,16	23,498	0,161	86,83	TruStrain™	289,7	1	6,28	11:35:23	9,13	148,04	37,00	0,210	0,47
16	0,0229	2,29	3,1435	134,77	25,101	0,186	115,46	TruStrain™	307,3	1	6,28	11:35:41	10,55	137,08	37,00	0,210	0,47
17	0,0329	3,29	4,119	122,19	26,58	0,218	151,29	TruStrain™	325,2	1	6,28	11:35:59	12,27	125,04	37,00	0,210	0,46
18	0,0473	4,73	5,3294	109,33	27,538	0,252	195,74	TruStrain™	342,9	1	6,28	11:36:16	14,14	112,74	37,00	0,210	0,46
19	0,0679	6,79	6,8121	96,369	27,878	0,289	250,2	TruStrain™	360,6	1	6,28	11:36:34	16,13	100,32	37,00	0,210	0,45
20	0,0975	9,75	8,5815	83,606	27,394	0,328	315,19	TruStrain™	377,9	1	6,28	11:36:51	18,14	87,98	37,00	0,210	0,45
21	0,14	14	10,66	71,522	26,33	0,368	391,54	TruStrain™	395,4	1	6,28	11:37:09	20,21	76,215	37,00	0,210	0,45
22	0,201	20,1	13,14	60,483	24,943	0,412	482,63	TruStrain™	412,9	1	6,28	11:37:26	22,41	65,424	37,00	0,210	0,44

Signature of operator: ______________________ Name: Date:
