# Supplementary material for: Structural and Viscoelastic Properties of Bacterial Cellulose Composites: Implications for Prosthetics
Source: Polymers (Basel). 2024 Nov 18;16(22):3200. doi: 10.3390/polym16223200 (PMC11597974; doi:10.3390/polym16223200)
Supplement: Supplementary file 1 [file polymers-16-03200-s001.zip › Cel_H_o┤_37_o│_PP50_S_F_0_25N_Amp_te _o╘o╤o▐_0_01_20%_f_1_Hz_08_08_2312_35_44.pdf]

Company:
Street:
City:

Report

Test | Info Cel_H_T_37_C_PP50_S_F_0.25N_Amp_te _рам_0_01_20%_f_1_Hz_08_08_23

Test created by operator: temp

Test creation date: 08.08.2023 12:27:52

Origin of project:

Rheometer: MCR 302 SN82961886

Measuring System: PP50/S SN79497

-

Sample | Info

Sample name:

Batch No.:

Description:

-

Result Data

LVE Limit:

LVE Proposal:

Flow Point TAU_y: tau = 0,02955 Pa; gamma = 0,01368 %; G' = 158,5 Pa
(if applicable)

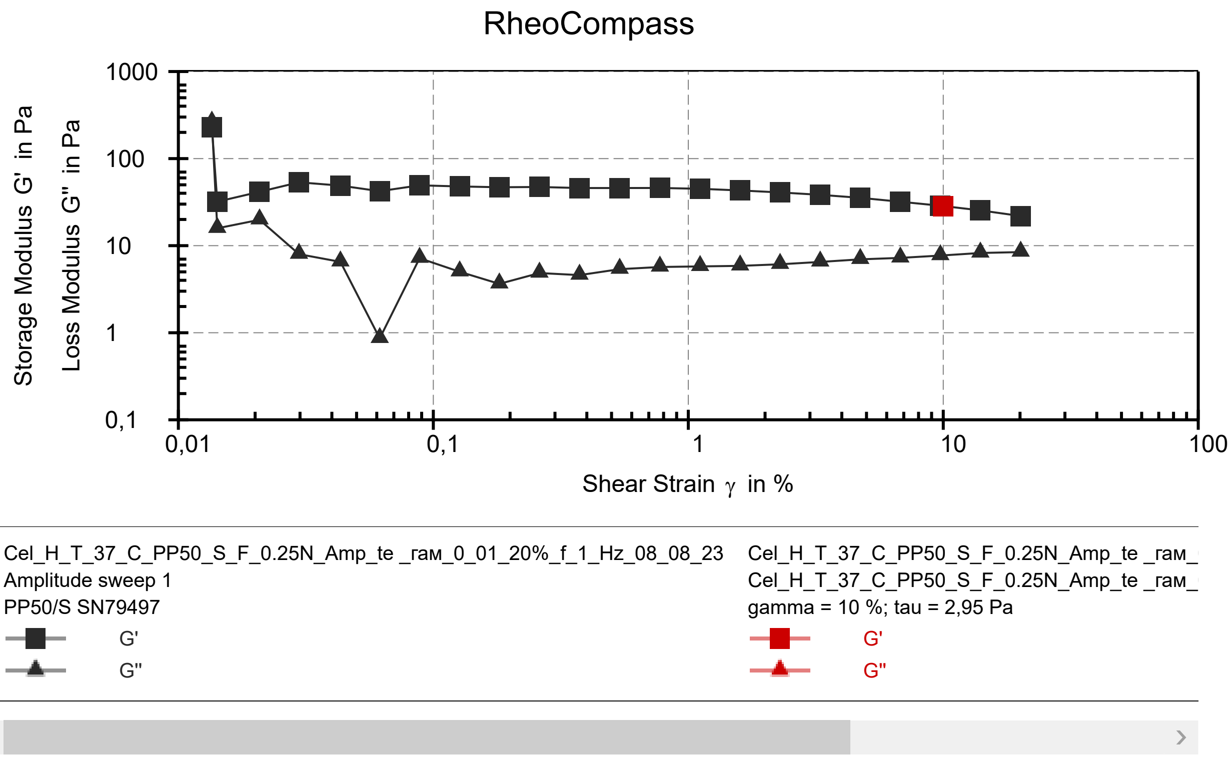

Anton Paar

Signature of operator: ________________________ Name: Date:

Company:
Street:
City:

Report

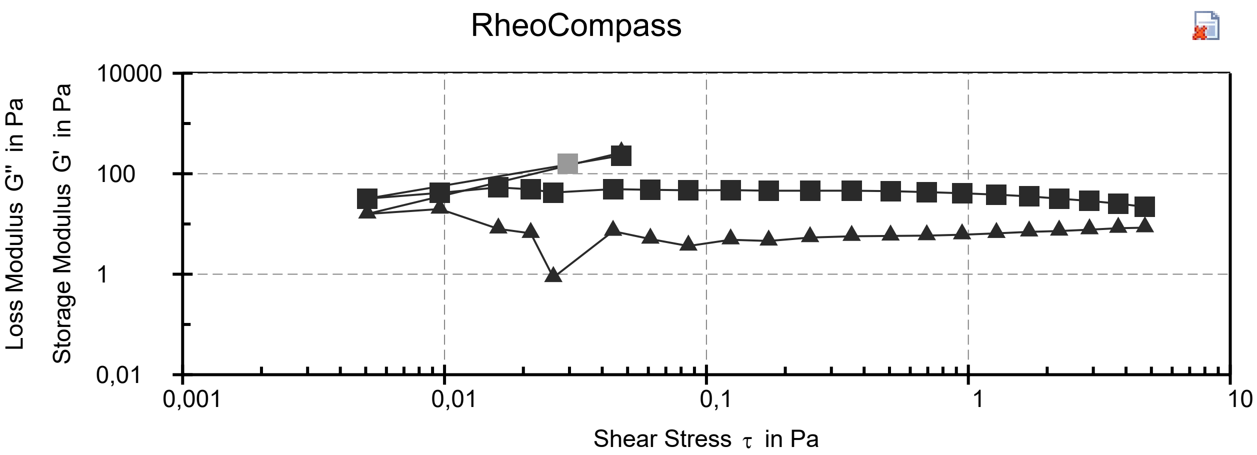

Anton Paar

Cel_H_T_37_C_PP50_S_F_0.25N_Amp_te _гам_0_01_20%_f_1_Hz_08_08_23, Amplitude sweep 1, Interval 1

Point No. Nº	Shear Str γ [1]	Shear Str γ [%]	Shear Str τ [Pa]	Storage I G' [Pa]	Loss Mod G'' [Pa]	Loss Fact tan(δ) [1]	Torque M [μN·m]	Status Stat	Time t [s]	Frequenc f [Hz]	Angular I ω [rad/s]	Time of D t_{abs}	Phase Sh δ [°]	Complex |G*| [Pa]	Temperat T [°C]	Gap d [mm]	Normal F F_N [N]
1	0,000135	0,0135	0,047338	230,55	262,97	1,141	1,7387	WMa,Tru Strain™	28,39	1	6,28	12:28:34	48,76	349,72	37,02	0,105	0,11
2	0,000142	0,0142	0,005073 1	31,992	15,832	0,495	0,18633	WMa,Tru Strain™	56,77	1	6,28	12:29:02	26,33	35,696	37,03	0,105	0,11
3	0,000208	0,0208	0,009588 4	41,57	19,817	0,477	0,35217	WMa,Tru Strain™	85,16	1	6,28	12:29:30	25,49	46,052	37,03	0,105	0,11
4	0,000297	0,0297	0,016051	53,407	7,9841	0,149	0,58954	TruStrain ™	112,9	1	6,28	12:29:58	8,50	54,001	37,02	0,105	0,11
5	0,000432	0,0432	0,021338	48,952	6,517	0,133	0,78371	TruStrain ™	138,3	1	6,28	12:30:24	7,58	49,384	37,02	0,105	0,11
6	0,000616	0,0616	0,026032	42,227	0,86122	0,020	0,95613	TruStrain ™	157	1	6,28	12:30:42	1,17	42,236	37,01	0,105	0,11
7	0,000883	0,0883	0,044	49,297	7,1774	0,146	1,6161	TruStrain ™	177,6	1	6,28	12:31:03	8,28	49,817	37,01	0,105	0,10
8	0,00127	0,127	0,061084	47,88	5,012	0,105	2,2436	TruStrain ™	195	1	6,28	12:31:20	5,98	48,141	37,01	0,105	0,10
9	0,00182	0,182	0,085322	46,864	3,6418	0,078	3,1338	TruStrain ™	212,3	1	6,28	12:31:38	4,44	47,005	37,01	0,105	0,10
10	0,00261	0,261	0,12372	47,188	4,8475	0,103	4,5442	TruStrain ™	230,2	1	6,28	12:31:55	5,87	47,437	37,00	0,105	0,10
11	0,00374	0,374	0,17281	45,923	4,5907	0,100	6,3472	TruStrain ™	247,3	1	6,28	12:32:13	5,71	46,152	37,00	0,105	0,10
12	0,00538	0,538	0,24871	45,904	5,3698	0,117	9,1349	TruStrain ™	264,6	1	6,28	12:32:30	6,67	46,217	37,00	0,105	0,10
13	0,00774	0,774	0,3585	45,973	5,6857	0,124	13,167	TruStrain ™	281,4	1	6,28	12:32:47	7,05	46,323	37,00	0,105	0,10
14	0,0111	1,11	0,50474	45,004	5,7778	0,128	18,539	TruStrain ™	299,4	1	6,28	12:33:05	7,32	45,373	37,00	0,105	0,10
15	0,016	1,6	0,6939	43,055	5,8599	0,136	25,486	TruStrain ™	317,2	1	6,28	12:33:23	7,75	43,452	37,00	0,105	0,10
16	0,0229	2,29	0,94647	40,865	6,1105	0,150	34,763	TruStrain ™	334,6	1	6,28	12:33:40	8,50	41,32	37,00	0,105	0,10
17	0,0329	3,29	1,2801	38,352	6,5029	0,170	47,018	TruStrain ™	351,7	1	6,28	12:33:57	9,62	38,899	37,00	0,105	0,10
18	0,0473	4,73	1,7063	35,427	6,9654	0,197	62,673	TruStrain ™	368,9	1	6,28	12:34:14	11,12	36,106	37,00	0,105	0,10
19	0,0679	6,79	2,2222	31,931	7,2394	0,227	81,617	TruStrain ™	386,7	1	6,28	12:34:32	12,77	32,741	37,00	0,105	0,10
20	0,0974	9,74	2,9009	28,767	7,6952	0,267	106,55	TruStrain ™	403,8	1	6,28	12:34:49	14,98	29,779	37,00	0,105	0,10
21	0,14	14	3,7358	25,423	8,2381	0,324	137,21	TruStrain ™	421,1	1	6,28	12:35:06	17,95	26,724	37,00	0,105	0,09
22	0,201	20,1	4,723	21,907	8,4384	0,385	173,47	TruStrain ™	438,6	1	6,28	12:35:24	21,07	23,476	37,00	0,105	0,09

Signature of operator: ________________________ Name: Date:
