# Supplementary material for: Structural and Viscoelastic Properties of Bacterial Cellulose Composites: Implications for Prosthetics
Source: Polymers (Basel). 2024 Nov 18;16(22):3200. doi: 10.3390/polym16223200 (PMC11597974; doi:10.3390/polym16223200)
Supplement: Supplementary file 1 [file polymers-16-03200-s001.zip › Cel_Re_H_o┤_37_o│_PP50_S_F_0_25N_Amp_te _o╘o╤o▐_0_01_20%_f_1_Hz_09_08_23_11_19_49.pdf]

Company:
Street:
City:

Report

Test | Info Cel_Re_H_T_37_C_PP50_S_F_0.25N_Amp_te _гам_0_01_20%_f_1_Hz_09_08_23_

Test created by operator: temp

Test creation date: 09.08.2023 11:10:47

Origin of project:

Rheometer: MCR 302 SN82961886

Measuring System: PP50/S SN79497

-

Sample | Info

Sample name:

Batch No.:

Description:

-

Result Data

LVE Limit:

LVE Proposal:

Flow Point TAU_y:
(if applicable)

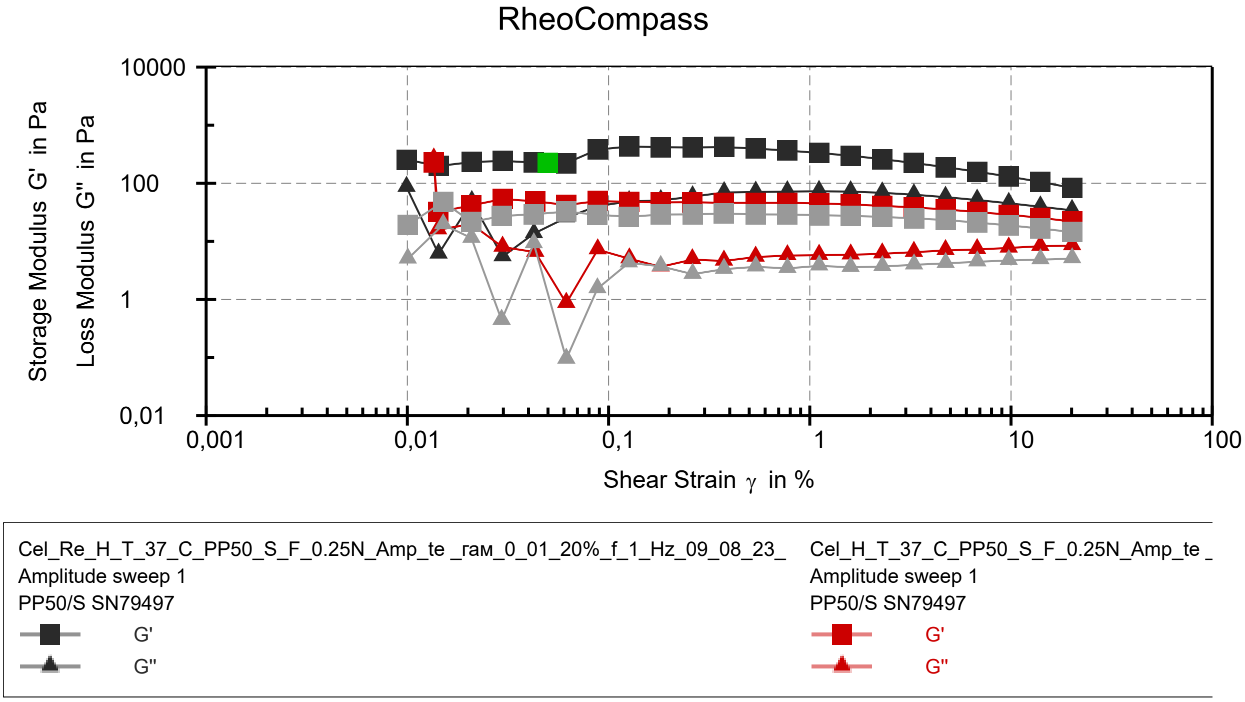

Anton Paar

Signature of operator: ________________________ Name: Date:

Company:
Street:
City:

Report

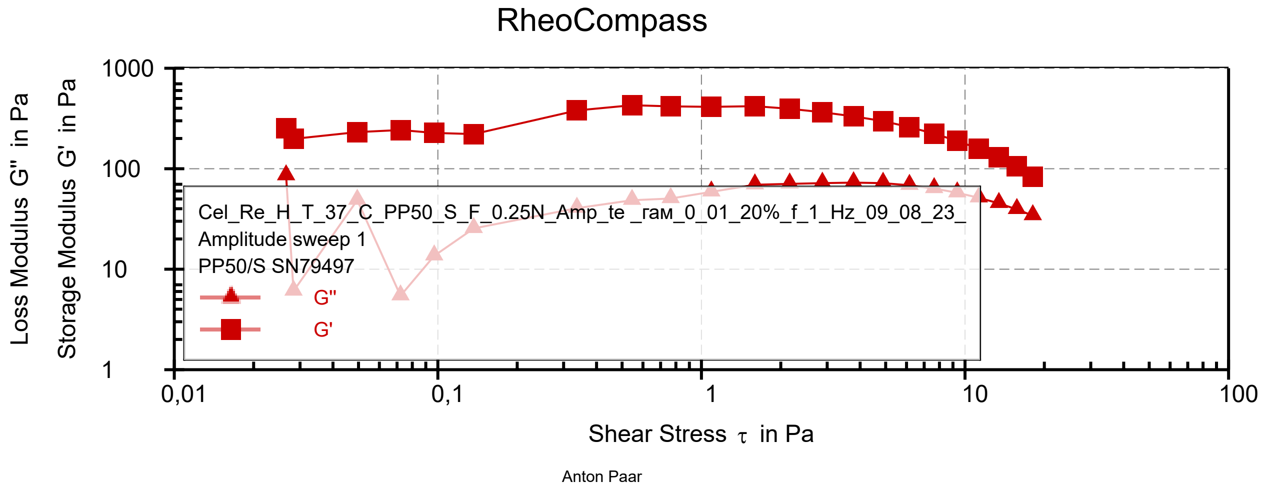

Cel_Re_H_T_37_C_PP50_S_F_0.25N_Amp_te _ram_0_01_20%_f_1_Hz_09_08_23_, Amplitude sweep 1, Interval 1

Point No №	Shear Str γ [1]	Shear Str γ [%]	Shear Str τ [Pa]	Storage I G' [Pa]	Loss Moc G'' [Pa]	Loss Fact tan(δ) [1]	Torque M [μN·m]	Status Stat	Time t [s]	Frequenc f [Hz]	Angular I ω [rad/s]	Time of [t_{abs}	Phase Sh δ [°]	Complex |G*| [Pa]	Temperat T [°C]	Gap d [mm]	Normal F F_N [N]
1	9,91E-05	0,00991	0,026559	254,13	85,304	0,336	0,97549	TruStrain™	21,25	1	6,28	11:11:21	18,56	268,07	37,04	0,126	0,22
2	0,000143	0,0143	0,028344	198,2	6,0648	0,031	1,041	MV-,TruS train™	48,94	1	6,28	11:11:48	1,75	198,29	37,03	0,126	0,19
3	0,000209	0,0209	0,049456	231,44	48,66	0,210	1,8165	TruStrain™	68,6	1	6,28	11:12:08	11,87	236,5	37,03	0,126	0,20
4	0,000297	0,0297	0,072165	242,73	5,4421	0,022	2,6506	MV-,TruS train™	86,99	1	6,28	11:12:27	1,28	242,79	37,02	0,126	0,20
5	0,000426	0,0426	0,096871	227,18	13,598	0,060	3,558	TruStrain™	105,7	1	6,28	11:12:45	3,43	227,58	37,02	0,126	0,20
6	0,000614	0,0614	0,13683	221,24	25,467	0,115	5,0258	TruStrain™	122,8	1	6,28	11:13:02	6,57	222,7	37,02	0,126	0,20
7	0,000881	0,0881	0,33694	380,09	40,522	0,107	12,375	TruStrain™	144,3	1	6,28	11:13:24	6,09	382,24	37,01	0,126	0,23
8	0,00126	0,126	0,54543	428,57	48,588	0,113	20,033	TruStrain™	161,8	1	6,28	11:13:41	6,47	431,31	37,01	0,126	0,23
9	0,00182	0,182	0,7645	417,55	50,529	0,121	28,079	TruStrain™	179,5	1	6,28	11:13:59	6,90	420,6	37,01	0,126	0,22
10	0,00261	0,261	1,0904	412,86	58,878	0,143	40,051	TruStrain™	197,1	1	6,28	11:14:17	8,12	417,04	37,01	0,126	0,22
11	0,00375	0,375	1,595	419,53	69,262	0,165	58,583	TruStrain™	214,4	1	6,28	11:14:34	9,37	425,21	37,01	0,126	0,22
12	0,00539	0,539	2,1616	395,03	70,67	0,179	79,394	TruStrain™	232,1	1	6,28	11:14:52	10,14	401,3	37,00	0,126	0,22
13	0,00773	0,773	2,8763	364,86	71,897	0,197	105,64	TruStrain™	249,2	1	6,28	11:15:09	11,15	371,88	37,00	0,126	0,21
14	0,0111	1,11	3,7774	332,08	72,757	0,219	138,74	TruStrain™	266,5	1	6,28	11:15:26	12,36	339,96	37,00	0,126	0,21
15	0,016	1,6	4,888	297,83	71,871	0,241	179,53	TruStrain™	284	1	6,28	11:15:43	13,57	306,38	37,00	0,126	0,21
16	0,0229	2,29	6,1553	259,81	68,455	0,263	226,08	TruStrain™	301,5	1	6,28	11:16:01	14,76	268,68	37,00	0,126	0,21
17	0,0329	3,29	7,6381	223,21	63,542	0,285	280,54	TruStrain™	318,9	1	6,28	11:16:18	15,89	232,08	37,00	0,126	0,21
18	0,0473	4,73	9,3442	189,17	57,639	0,305	343,2	TruStrain™	336,6	1	6,28	11:16:36	16,95	197,76	37,00	0,126	0,20
19	0,0679	6,79	11,281	158,08	51,408	0,325	414,35	TruStrain™	353,7	1	6,28	11:16:53	18,01	166,23	37,00	0,126	0,20
20	0,0975	9,75	13,446	130,36	45,224	0,347	493,87	TruStrain™	370,8	1	6,28	11:17:10	19,13	137,98	37,00	0,126	0,19
21	0,14	14	15,733	105,3	39,374	0,374	577,87	TruStrain™	388,6	1	6,28	11:17:28	20,50	112,42	37,00	0,126	0,18
22	0,201	20,1	18,081	83,212	34,178	0,411	664,1	TruStrain™	405,6	1	6,28	11:17:45	22,33	89,958	37,00	0,126	0,17

Signature of operator: ______________________ Name: Date:
