# Supplementary material for: Structural and Viscoelastic Properties of Bacterial Cellulose Composites: Implications for Prosthetics
Source: Polymers (Basel). 2024 Nov 18;16(22):3200. doi: 10.3390/polym16223200 (PMC11597974; doi:10.3390/polym16223200)
Supplement: Supplementary file 1 [file polymers-16-03200-s001.zip › Cel_static_o┤_37_o│_PP50_S_F_0_25N_Amp_te _o╘o╤o▐_0_01_20%_f_1_Hz_22_09_23_12_30_09.pdf]

Company:
Street:
City:

Report

Test | Info

**Test | Info**	Cel_static_T_37_C_PP50_S_F_0_25N_Amp_te _гам_0_01_20%_f_1_Hz_22_09_23_
Test created by operator:	temp
Test creation date:	22.09.2023 12:18:05
Origin of project:	
Rheometer:	MCR 302 SN82961886
Measuring System:	PP50/S SN79497

-

Sample | Info

Sample name:
Batch No.:

Description:

-

Result Data

LVE Limit:
LVE Proposal:

Flow Point TAU_y: (if applicable) tau = 0,01905 Pa; gamma = 0,01198 %; G' = 152,1 Pa

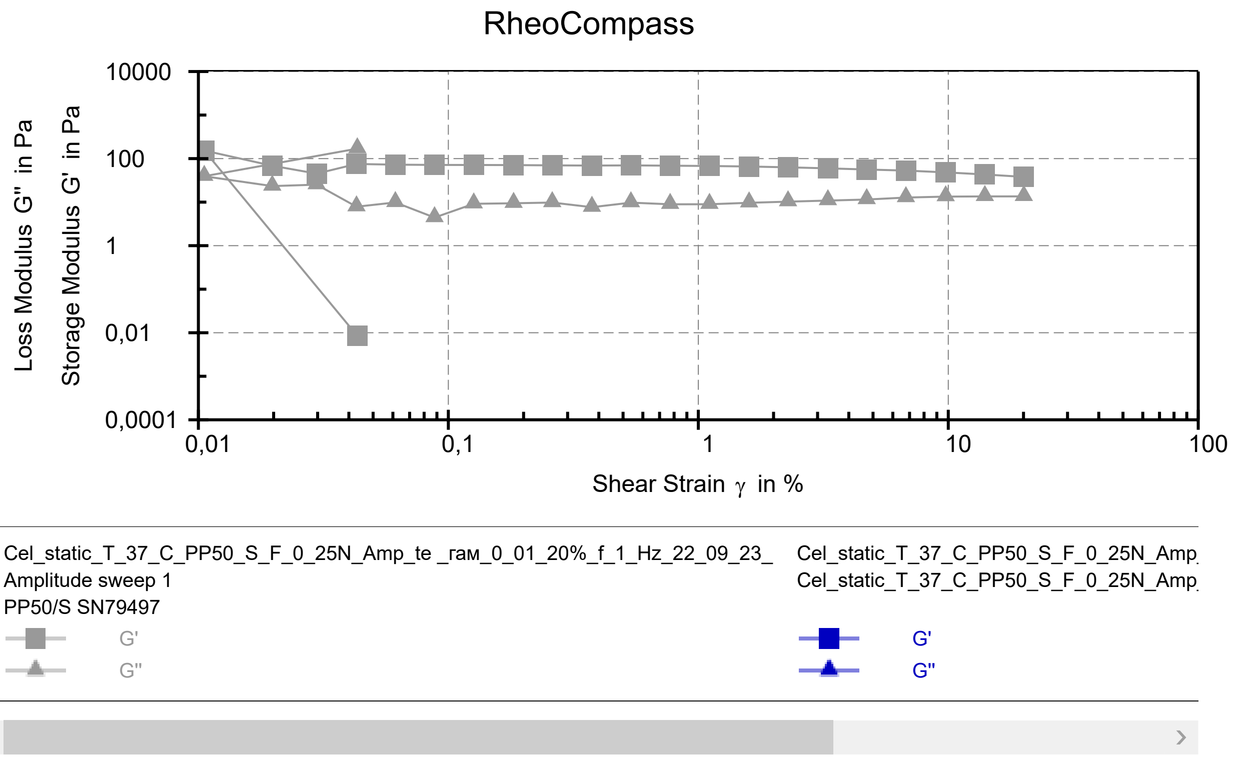

Anton Paar

Signature of operator: ______________________ Name: Date:

Company:
Street:
City:

Report

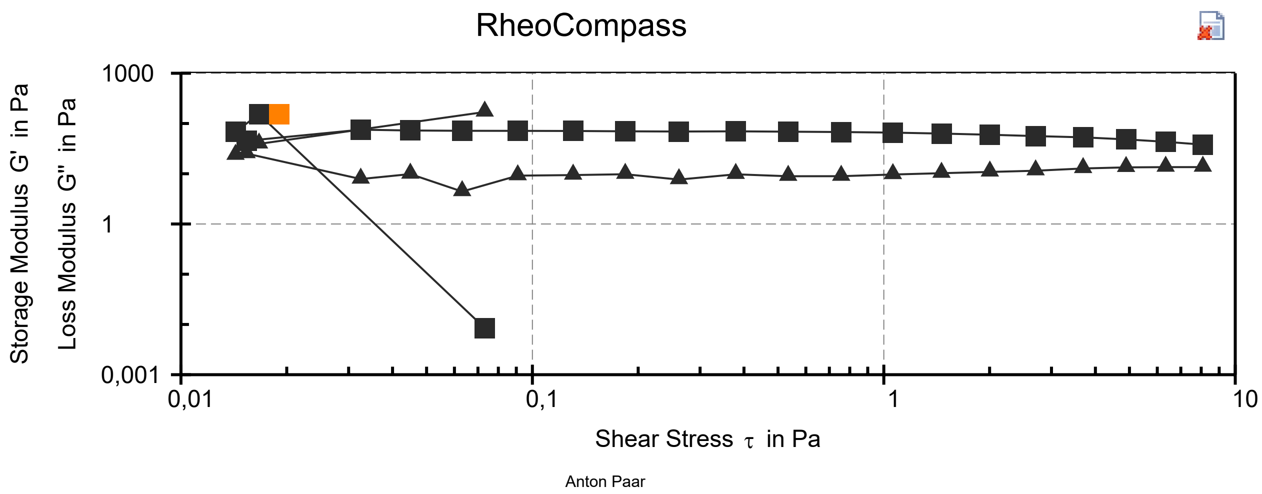

Cel_static_T_37_C_PP50_S_F_0_25N_Amp_te _гам_0_01_20%_f_1_Hz_22_09_23_, Amplitude sweep 1, Interval 1

Point No Nº	Shear Str γ [1]	Shear Str γ [%]	Shear Str τ [Pa]	Storage I G' [Pa]	Loss Moc G'' [Pa]	Loss Fact tan(δ) [1]	Torque M [μN·m]	Status Stat	Time t [s]	Frequenc f [Hz]	Angular I ω [rad/s]	Time of [t_{abs}	Phase Sh δ [°]	Complex |G*| [Pa]	Temperat T [°C]	Gap d [mm]	Normal F F_N [N]
1	0,000432	0,0432	0,073185	0,008463 1	169,26	20000,00 0	2,688	ME-,WM a,taD,Tru Strain™	28,39	1	6,28	12:18:45	90,00	169,26	37,03	0,098	0,48
2	0,000106	0,0106	0,016681	153,09	39,288	0,257	0,61267	WMa,Tru Strain™	56,77	1	6,28	12:19:13	14,39	158,05	37,03	0,098	0,43
3	0,000198	0,0198	0,014299	68,516	23,366	0,341	0,52518	WMa,Tru Strain™	85,16	1	6,28	12:19:41	18,83	72,391	37,03	0,098	0,41
4	0,000297	0,0297	0,01537	45,15	25,196	0,558	0,56453	TruStrain ™	102,8	1	6,28	12:19:59	29,16	51,704	37,02	0,098	0,39
5	0,000429	0,0429	0,03248	75,294	7,8445	0,104	1,193	TruStrain ™	123,4	1	6,28	12:20:20	5,95	75,701	37,02	0,098	0,37
6	0,000614	0,0614	0,044944	72,567	9,8444	0,136	1,6508	TruStrain ™	141,2	1	6,28	12:20:37	7,73	73,231	37,01	0,098	0,36
7	0,000881	0,0881	0,06312	71,538	4,4205	0,062	2,3184	TruStrain ™	159	1	6,28	12:20:55	3,54	71,674	37,01	0,098	0,35
8	0,00126	0,126	0,090856	71,301	9,1548	0,128	3,3371	TruStrain ™	174,8	1	6,28	12:21:11	7,32	71,886	37,01	0,098	0,34
9	0,00182	0,182	0,1306	70,96	9,4079	0,133	4,7967	TruStrain ™	193,7	1	6,28	12:21:30	7,55	71,58	37,01	0,098	0,33
10	0,0026	0,26	0,18375	69,877	9,7734	0,140	6,749	TruStrain ™	211	1	6,28	12:21:47	7,96	70,557	37,01	0,098	0,32
11	0,00375	0,375	0,26125	69,184	7,6485	0,111	9,5955	TruStrain ™	228,6	1	6,28	12:22:05	6,31	69,605	37,00	0,098	0,31
12	0,00538	0,538	0,37903	69,834	9,7413	0,139	13,922	TruStrain ™	245,5	1	6,28	12:22:22	7,94	70,511	37,00	0,098	0,29
13	0,00772	0,772	0,53442	68,693	8,9208	0,130	19,629	TruStrain ™	262,3	1	6,28	12:22:38	7,40	69,27	37,00	0,098	0,29
14	0,0111	1,11	0,75631	67,664	8,9357	0,132	27,779	TruStrain ™	279,5	1	6,28	12:22:56	7,52	68,251	37,00	0,098	0,28
15	0,016	1,6	1,0613	65,772	9,6525	0,147	38,981	TruStrain ™	296,7	1	6,28	12:23:13	8,35	66,476	37,00	0,098	0,27
16	0,0229	2,29	1,4593	63,019	10,254	0,163	53,597	TruStrain ™	313,9	1	6,28	12:23:30	9,24	63,848	37,00	0,098	0,26
17	0,033	3,3	2,003	59,73	10,857	0,182	73,569	TruStrain ™	332,1	1	6,28	12:23:48	10,30	60,709	37,00	0,098	0,25
18	0,0472	4,72	2,7037	56,147	11,473	0,204	99,303	TruStrain ™	349,2	1	6,28	12:24:05	11,55	57,308	37,00	0,098	0,24
19	0,0677	6,77	3,6904	53,01	12,732	0,240	135,54	TruStrain ™	366,5	1	6,28	12:24:23	13,51	54,517	37,00	0,098	0,24
20	0,0977	9,77	4,8899	48,251	13,384	0,277	179,6	TruStrain ™	384,2	1	6,28	12:24:40	15,50	50,073	37,00	0,098	0,24
21	0,14	14	6,3393	43,184	13,534	0,313	232,84	TruStrain ™	402,2	1	6,28	12:24:58	17,40	45,255	37,00	0,098	0,23
22	0,201	20,1	8,0874	37,975	13,544	0,357	297,04	TruStrain ™	419,6	1	6,28	12:25:16	19,63	40,318	37,00	0,098	0,22

Signature of operator: ______________________ Name: Date:
