# Supplementary material for: Structural and Viscoelastic Properties of Bacterial Cellulose Composites: Implications for Prosthetics
Source: Polymers (Basel). 2024 Nov 18;16(22):3200. doi: 10.3390/polym16223200 (PMC11597974; doi:10.3390/polym16223200)
Supplement: Supplementary file 1 [file polymers-16-03200-s001.zip › Cel_S_o┤_37_o│_PP50_S_F_0_25N_Amp_te _o╘o╤o▐_0_01_20%_f_1_Hz_08_08_2315_57_21.pdf]

Company:
Street:
City:

Report

Test | Info Cel_S_T_37_C_PP50_S_F_0_25N_Amp_te _гам_0_01_20%_f_1_Hz_08_08_23

Test created by operator: temp

Test creation date: 08.08.2023 15:49:10

Origin of project:

Rheometer: MCR 302 SN82961886

Measuring System: PP50/S SN79497

-

Sample | Info

Sample name:

Batch No.:

Description:

-

Result Data

LVE Limit:

LVE Proposal:

Flow Point TAU_y:
(if applicable)

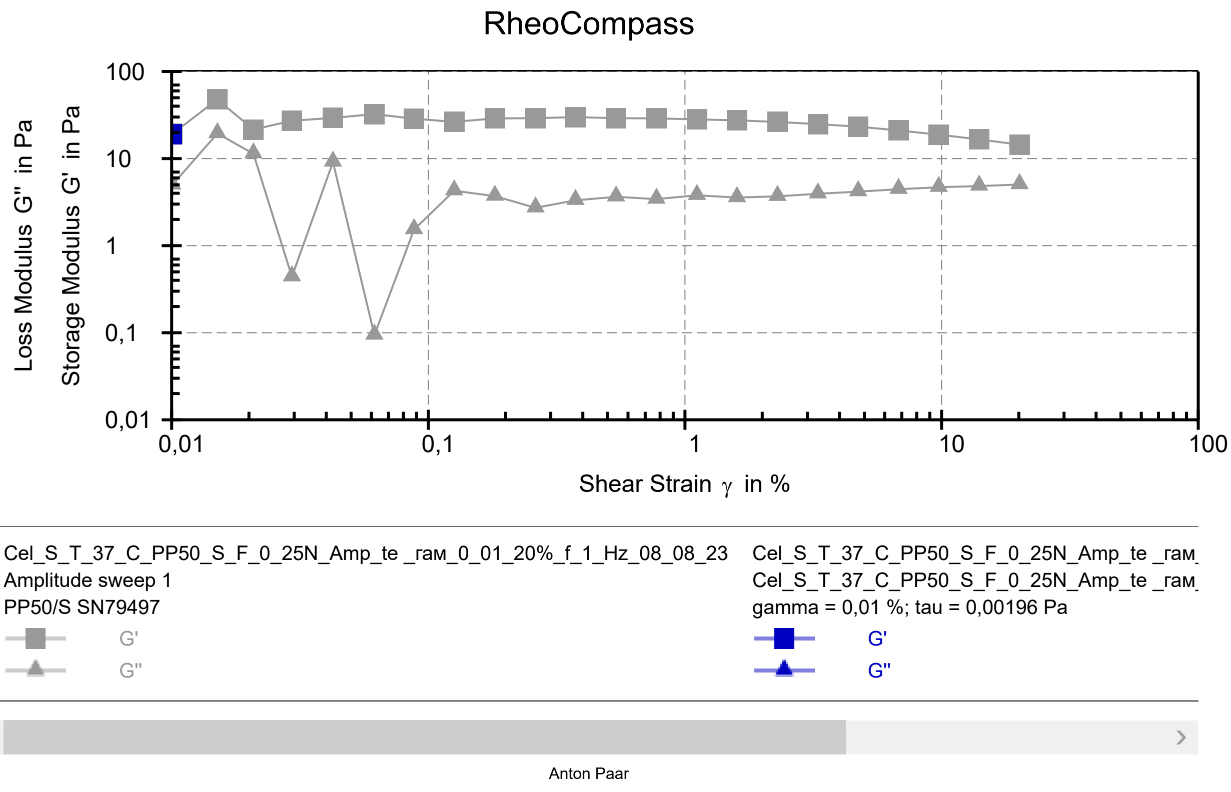

Signature of operator: ______________________ Name: Date:

Company:
Street:
City:

Report

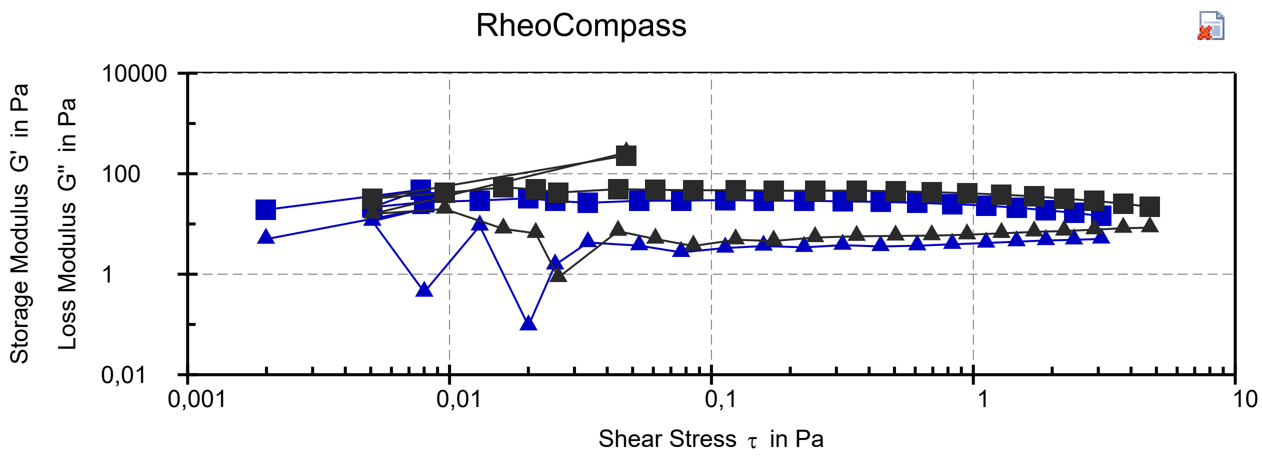

Cel_S_T_37_C_PP50_S_F_0_25N_Amp_te _гам_0_01_20%_f_1_Hz_08_08_23, Amplitude sweep 1, Interval 1

Point No Nº	Shear Str γ [1]	Shear Str γ [%]	Shear Str τ [Pa]	Storage I G' [Pa]	Loss Moc G'' [Pa]	Loss Fact tan(δ) [1]	Torque M [µN·m]	Status Stat	Time t [s]	Frequenc f [Hz]	Angular I ω [rad/s]	Time of I t_{abs}	Phase Sh δ [°]	Complex |G*| [Pa]	Temperat T [°C]	Gap d [mm]	Normal F F_N [N]
1	0,0001	0,01	0,0019908	19,171	4,9857	0,260	0,07312	WMa,TruStrain™	28,39	1	6,28	15:49:51	14,58	19,809	37,04	0,076	0,02
2	0,000151	0,0151	0,0077798	47,825	19,42	0,406	0,28574	WMa,TruStrain™	56,77	1	6,28	15:50:19	22,10	51,617	37,03	0,076	0,01
3	0,000208	0,0208	0,0050733	21,555	11,366	0,527	0,18634	WMa,TruStrain™	85,16	1	6,28	15:50:48	27,80	24,368	37,03	0,076	0,01
4	0,000294	0,0294	0,0080068	27,22	0,43983	0,016	0,29408	WMa,TruStrain™	113,6	1	6,28	15:51:16	0,93	27,224	37,02	0,076	0,00
5	0,000425	0,0425	0,013044	29,301	9,1295	0,312	0,4791	TruStrain™	138,6	1	6,28	15:51:41	17,31	30,691	37,01	0,076	0,00
6	0,000617	0,0617	0,02001	32,454	0,094124	0,003	0,73496	MV-,TruStrain™	161,2	1	6,28	15:52:04	0,17	32,454	37,01	0,076	-0,01
7	0,00088	0,088	0,025294	28,701	1,5318	0,053	0,92902	TruStrain™	184,6	1	6,28	15:52:27	3,05	28,742	37,01	0,076	-0,01
8	0,00126	0,126	0,033752	26,416	4,2753	0,162	1,2397	TruStrain™	202,5	1	6,28	15:52:45	9,19	26,76	37,00	0,076	-0,01
9	0,00182	0,182	0,052988	28,933	3,7079	0,128	1,9462	TruStrain™	219	1	6,28	15:53:01	7,30	29,17	37,00	0,076	-0,02
10	0,00262	0,262	0,076586	29,14	2,7204	0,093	2,8129	TruStrain™	237,3	1	6,28	15:53:20	5,33	29,267	37,01	0,076	-0,02
11	0,00375	0,375	0,1129	29,954	3,3194	0,111	4,1466	TruStrain™	255,2	1	6,28	15:53:38	6,32	30,138	37,00	0,076	-0,02
12	0,00539	0,539	0,15808	29,099	3,6373	0,125	5,8061	TruStrain™	272,2	1	6,28	15:53:55	7,12	29,326	37,00	0,076	-0,03
13	0,00774	0,774	0,22584	28,966	3,4257	0,118	8,2948	TruStrain™	289,5	1	6,28	15:54:12	6,74	29,168	37,00	0,076	-0,03
14	0,0111	1,11	0,31666	28,19	3,787	0,134	11,631	TruStrain™	307,5	1	6,28	15:54:30	7,65	28,443	37,00	0,076	-0,04
15	0,016	1,6	0,44238	27,503	3,5698	0,130	16,248	TruStrain™	325,1	1	6,28	15:54:48	7,40	27,734	37,00	0,076	-0,04
16	0,0229	2,29	0,61046	26,349	3,6861	0,140	22,422	TruStrain™	342,9	1	6,28	15:55:05	7,96	26,605	37,00	0,076	-0,04
17	0,033	3,3	0,83057	24,888	3,9412	0,158	30,506	TruStrain™	360,7	1	6,28	15:55:23	9,00	25,198	37,00	0,076	-0,05
18	0,0473	4,73	1,1183	23,255	4,1774	0,180	41,074	TruStrain™	378,4	1	6,28	15:55:41	10,18	23,627	37,00	0,076	-0,06
19	0,068	6,8	1,462	21,041	4,4447	0,211	53,696	TruStrain™	396,1	1	6,28	15:55:58	11,93	21,506	37,00	0,076	-0,06
20	0,0975	9,75	1,8923	18,839	4,6875	0,249	69,503	TruStrain™	413,6	1	6,28	15:56:16	13,97	19,413	37,00	0,076	-0,07
21	0,14	14	2,4239	16,617	4,8373	0,291	89,028	TruStrain™	431,2	1	6,28	15:56:34	16,23	17,307	37,00	0,076	-0,08
22	0,201	20,1	3,0767	14,434	5,0336	0,349	113	TruStrain™	448,7	1	6,28	15:56:51	19,23	15,287	37,00	0,076	-0,08

Signature of operator: ______________________ Name: Date:
